# Dynamic NFIX Promoter Usage Shapes Stem and Committed Myogenic States

**DOI:** 10.3390/ijms27167367

**Published:** 2026-08-18

**Authors:** Gabriele Rovetta, Giulia Ferrari, Mirko Ronzio, Alberto Gallo, Raffaele Epis, Chiara Bonfanti, Diletta Dolfini, Giorgia Careccia, Graziella Messina

**Affiliations:** Department of Biosciences, University of Milan, 20133 Milan, Italy; gabriele.rovetta@unimi.it (G.R.); giulia.ferrari3@unimi.it (G.F.); mirko.ronzio@unimi.it (M.R.); alberto.gallo1@unimi.it (A.G.); raffaele.epis@unimi.it (R.E.); chiara.bonfanti@unimi.it (C.B.); graziella.messina@unimi.it (G.M.)

**Keywords:** NFIX, skeletal muscle, myogenesis, muscle regeneration, satellite cells, alternative promoters, epigenetic regulation, transcript isoforms

## Abstract

Skeletal muscle development and regeneration rely on coordinated transcriptional programs controlled by muscle stem cells and their progeny. Nuclear Factor I X (NFIX) plays essential roles in fetal myogenesis and adult muscle regeneration. However, the mechanisms regulating its transcription and isoform expression remain unclear. Here, integrating multi-omics analyses with in vitro functional assays, we define the transcriptional and epigenetic landscape controlling Nfix expression during skeletal muscle development and regeneration. We show that *Nfix* promoter usage is dynamically regulated according to myogenic cell state: promoter 2 is associated with quiescent and stem-like states, whereas promoter 1 is activated during myogenic commitment and regeneration. These programs are accompanied by differential enhancer accessibility and DNA methylation changes within the *Nfix* locus, indicating coordinated epigenetic regulation. We further demonstrate that alternative promoter usage and exon 7/9 splicing generate distinct NFIX isoforms affecting myoblast proliferation and fusion, while transcriptomic profiling identified NFIX-dependent networks involved in muscle structure and metabolism. Overall, our findings uncover multilayered mechanisms controlling Nfix expression and identify promoter- and isoform-specific NFIX programs associated with stem, regenerative, and differentiated myogenic states. These results provide a molecular framework for future therapeutic strategies targeting NFIX in muscle diseases.

## 1. Introduction

Nuclear Factor I X (NFIX) is a transcription factor (TF) belonging to the NFI family, which also includes NFIA, NFIB, and NFIC [1]. In skeletal muscle, NFIX plays fundamental roles during both developmental myogenesis and adult muscle regeneration [2,3].

The *Nfix* gene is located on the reverse strand of chromosome 8qC3, and displays a complex regulatory architecture characterized by multiple promoters, intragenic enhancers, and alternative splicing events (Mouse, GRCm38/mm10). Among the five annotated promoters, promoters 1 and 2 are the most extensively characterized and generate the majority of Nfix transcripts [4]. Nfix transcripts are composed of 11 exons: exon 1 encodes the 5′ untranslated region (5′UTR), exon 2 gives rise to the N-terminal and MH1 DNA-binding domains [5], exons 4–10 contribute to the C-terminal region, whereas exon 11 encodes the 3′ untranslated region (3′UTR). Notably, exons 7 and 9 undergo alternative splicing, thereby contributing to the generation of distinct Nfix transcript variants [6].

In addition to its complex architecture, the murine *Nfix* locus harbors nine intragenic enhancers involved in the regulation of Nfix transcription. Enhancers 1–4 are positioned between promoters 1 and 2, while enhancers 5–9 are located downstream of promoter 2. Through long-range chromatin interactions with a regulatory region situated between promoters 1 and 2, these enhancers contribute to the dynamic chromatin remodeling of the *Nfix* locus, likely influencing Nfix transcriptional regulation [7].

Functionally, NFIX is a key regulator of skeletal muscle development, particularly during the transition from embryonic (primary, E10.5–E12.5 in mouse) to fetal (secondary, E14.5–E17.5 in mouse) myogenesis [8]. During this developmental switch, NFIX represses embryonic transcriptional programs while activating fetal-specific genes, thereby promoting muscle maturation [2]. NFIX also contributes to muscle fiber-type specification through the modulation of Sox6 activity [7]. Mechanistically, Nfix expression during fetal myogenesis is sustained by JunB downstream of the RhoA/ROCK–ERK signaling axis [9]. Upon activation, NFIX promotes the transcription of fetal-specific genes, such as β-enolase (Eno3), Muscle Creatine Kinases (MCKs), Protein Kinase C θ (PKCθ) and fast MyHC, while repressing embryonic ones, such as slow MyHC. Consistently, Nfix-deficient mice display impaired skeletal muscle development, reduced fetal size, and sarcomeric disorganization, whereas NFIX overexpression accelerates fetal-like muscle maturation [2]. Despite these findings, the molecular mechanisms regulating *Nfix* promoter activity and transcript expression during muscle development remain poorly characterized.

Beyond developmental myogenesis, NFIX also regulates adult skeletal muscle regeneration, a process that partially recapitulates developmental transcriptional programs. Following muscle injury, satellite cells (SCs) become activated, proliferate, and differentiate into myoblasts that fuse to regenerate damaged myofibers. In this context, NFIX controls the proper timing of SC differentiation and regenerative progression. Indeed, *Nfix*-deficient SCs display reduced expression of the myogenic regulators Myod and Myogenin, while *Nfix*^−/−^ mice exhibit reduced body size and decreased myofiber cross-sectional area [3]. NFIX additionally acts in macrophages, where it promotes the transition from pro-inflammatory to anti-inflammatory phenotypes, thereby supporting inflammation resolution and tissue repair [10]. However, the mechanisms regulating *Nfix* promoter activity and transcript expression during SC activation and muscle regeneration remain largely unknown.

Recently, NFIX also emerged as a potential therapeutic target in several diseases. In skeletal muscle, NFIX modulation has been associated with beneficial effects in Muscular Dystrophies [11,12,13] and Myotonic Dystrophy [14,15,16]. Moreover, altered NFIX activity has been implicated in additional pathological contexts, including Marshall–Smith Syndrome [17], Medulloblastoma [18], breast cancer [19], and lung cancer [20]. These observations highlight the importance of understanding the molecular mechanisms controlling *Nfix* expression across physiological and pathological conditions.

Despite the established role of NFIX in skeletal muscle biology, several aspects of its regulation remain unclear. In particular, it is still unknown how distinct *Nfix* promoters are differentially regulated across myogenic states, whether promoter usage contributes to the generation of functionally distinct isoforms, and which transcriptional networks are controlled by NFIX during skeletal muscle development and regeneration. Addressing these questions is essential to achieve a more comprehensive understanding of NFIX function and to support the development of NFIX-targeted therapeutic approaches.

Here, by integrating multi-omics data analysis with in vitro functional approaches, we define the transcriptional landscape controlling *Nfix* gene expression in skeletal muscle. We identify promoter-specific regulatory programs associated with stem and committed myogenic states, uncover epigenetic and post-transcriptional mechanisms shaping Nfix isoform expression, and characterize NFIX-dependent transcriptional networks involved in muscle structure and metabolism. Overall, these findings provide new insights into the regulatory network controlling Nfix expression in skeletal muscle and establish a molecular framework for future translational strategies targeting NFIX-related muscle diseases.

## 2. Results

### 2.1. Distinct Nfix Promoters Are Associated with Stem and Committed Myogenic States

**Figure 1:**
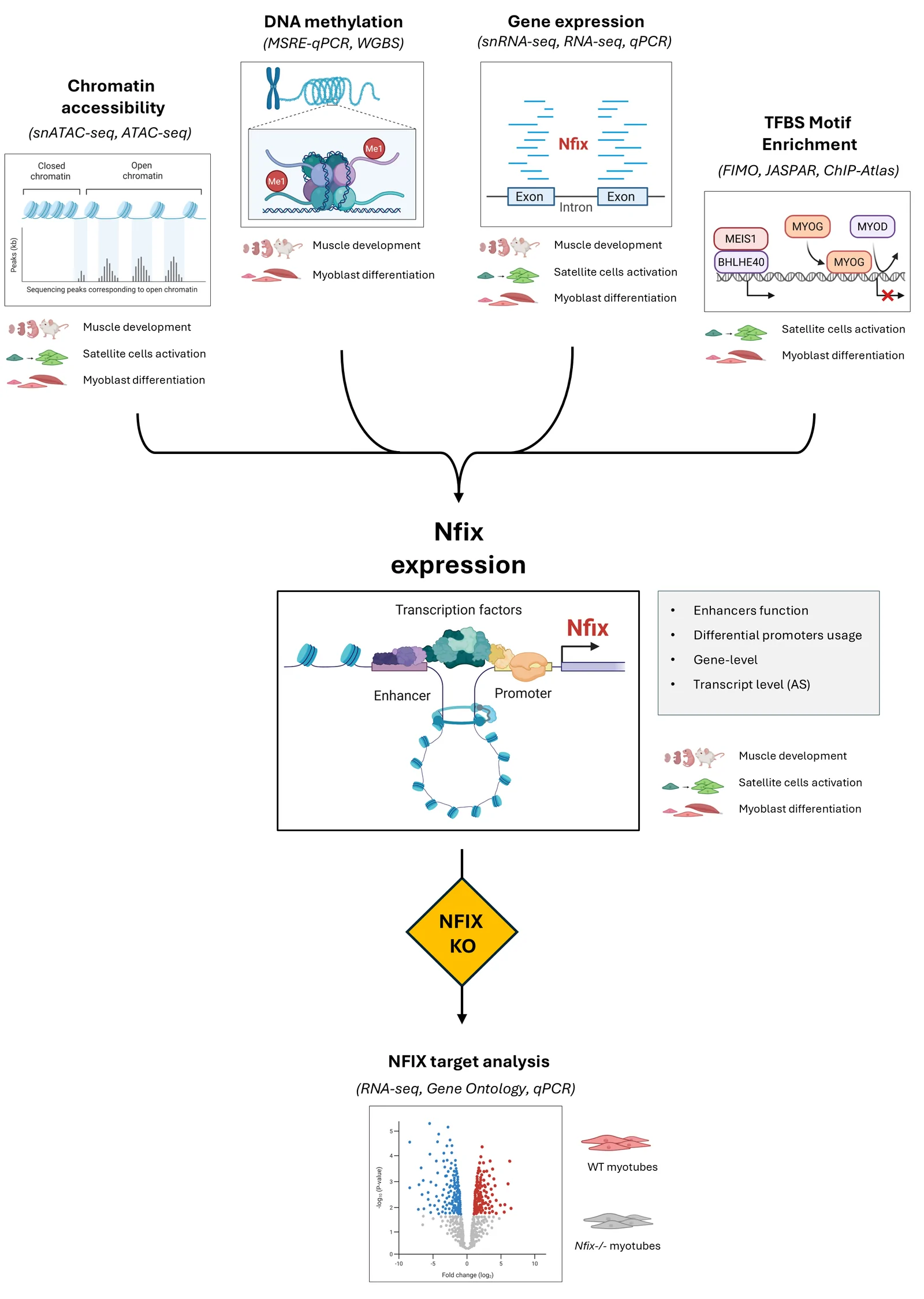
Workflow of the analysis of Nfix expression. Schematic representation of the integrated bioinformatic and experimental approaches used to investigate the epigenetic, transcriptional, and post-transcriptional regulation of Nfix during skeletal muscle development, satellite cell activation, and myoblast differentiation, together with the identification of NFIX-regulated target genes.

**Figure 2:**
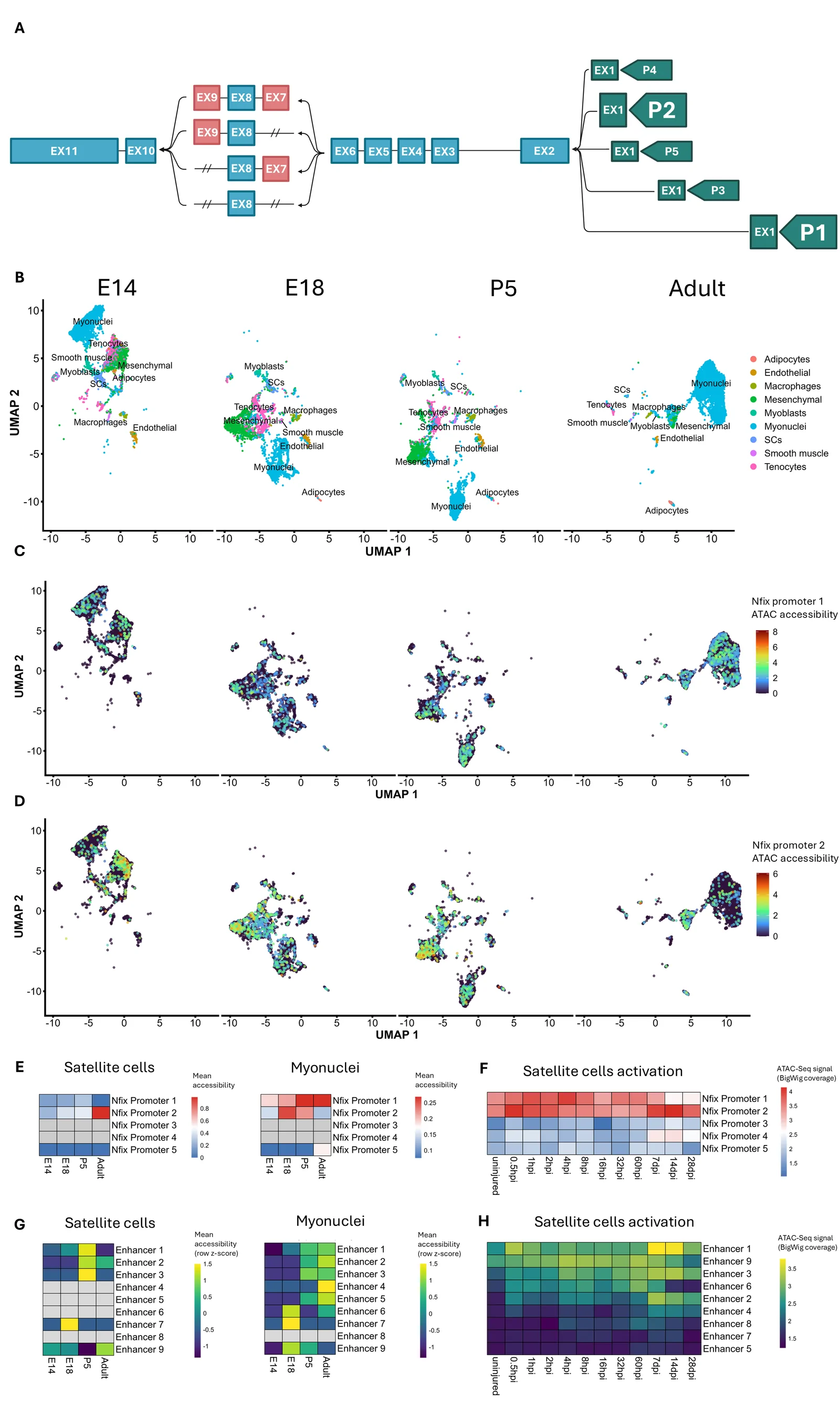
*Nfix* promoters are differentially accessible in skeletal muscle development and regeneration. (**A**) Schematic structure of the murine *Nfix* gene. Promoters and exon 1 are indicated in green, common exons in blue, and alternatively spliced exons in red. P: promoter, EX: exon. (**B**) Different cell populations, (**C**) *Nfix* promoter 1 accessibility (Tn5 insertions), and (**D**) *Nfix* promoter 2 accessibility (Tn5 insertions) during skeletal muscle development represented through UMAP graphs (E14: embryonal; E18: fetal; P5: juvenile; Adult: adult). (**E**) *Nfix* promoters accessibility during satellite cells and myonuclei development and (**F**) during satellite cell activation upon murine muscle injury. Values are shown as mean accessibility; NA values are indicated in grey. (**G**) *Nfix* enhancer accessibility during satellite cell and myonuclei development, and (**H**) satellite cell activation upon murine muscle injury, based on BigWig coverage data.

To investigate the transcriptional regulation of Nfix in skeletal muscle, we first examined chromatin accessibility at the *Nfix* locus during murine muscle development and regeneration (Figure 1). Analysis of published snATAC-Seq data during skeletal muscle development (GSE211542) in embryonal (E14), fetal (E18), juvenile (P5), and adult (Adult) muscles revealed differential *Nfix* promotersaccessibility across distinct cell types and developmental stages (Figure 2A–D and Figure S1A,B). *Nfix* promoters 1 and 2, which are the most characterized *Nfix* promoters (Figure 2A), display opposite chromatin accessibility profiles during skeletal muscle development. Specifically, *Nfix* promoter 1 was predominantly accessible in adipocytes, myonuclei, and endothelial cells, whereas *Nfix* promoter 2 showed higher accessibility in tenocytes, satellite cells (SCs), mesenchymal cells, and macrophages (Figure 2A–D and Figure S1A,B).

Focusing on myogenic cell types (SCs and myonuclei), we observed distinct accessibility patterns for the two *Nfix* promoters throughout skeletal muscle development. *Nfix* promoter 1 remained inaccessible during SC development. On the contrary, *Nfix* promoter 2 progressively increased its accessibility from E14 to adult SCs, where chromatin remained highly accessible compared with *Nfix* promoter 1 (Figure 2E). In addition, in myonuclei, both *Nfix* promoters 1 and 2 became accessible in the embryo-fetal switch (E14 to E18), but their accessibility profiles diverged at later developmental stages in P5 and adult myonuclei. Specifically, chromatin accessibility at *Nfix* promoter 1 progressively increased, whereas accessibility at *Nfix* promoter 2 gradually decreased. Consequently, in adult myonuclei, *Nfix* promoter 1 was more accessible than *Nfix* promoter 2, while in adult SCs, *Nfix* promoter 1 was completely closed compared to *Nfix* promoter 2 (Figure 2C–E).

The dynamic changes in chromatin accessibility at *Nfix* promoters during SC and myonuclei development prompted us to investigate *Nfix* promoters states also during adult SC activation upon injury. To address this, we analyzed BigWig files derived from bulk ATAC-Seq (GSE189044) on Pax7^GFP+^ cells isolated at different time points after murine muscle injury (uninjured, 0.5 hpi, 1 hpi, 2 hpi, 4 hpi, 8 hpi, 16 hpi, 32 hpi, 60 hpi, 7 dpi, 14 dpi, and 28 dpi). As expected, *Nfix* promoters 1 and 2 were the most accessible regulatory regions compared to the other transcription start sites within the *Nfix* gene (*Nfix* promoters 3, 4, and 5) (Figure 2F). Moreover, in quiescent or quiescent-like SCs (uninjured, 0.5 hpi, 7 dpi, 14 dpi, and 28 dpi) (Supplementary Figure S1C), *Nfix* promoter 2 displayed higher chromatin accessibility than *Nfix* promoter 1, while *Nfix* promoter 1 became more accessible in activated SCs (1 hpi, 2 hpi, 4 hpi, 8 hpi, 16 hpi, 32 hpi, 60 hpi) (Figure 2F).

To identify mechanisms underlying the dynamic chromatin accessibility of *Nfix* promoters 1 and 2 in muscle development and SC activation, we investigated intragenic enhancer chromatin accessibility within the *Nfix* locus. To do that, we looked at the chromatin state of *Nfix* intragenic enhancers, identified through an enhancer-gene map from the ENCODE3 project [21]. We examined enhancer accessibility in E14, E18, P5, adult SCs, and myonuclei during muscle development, as well as SC activation at different time points after acute injury. This analysis revealed two spatially distinct clusters of intragenic *Nfix* enhancers. The first group, including enhancers 1 to 4, is located between *Nfix* promoters 1 and 2, while the second group, which includes enhancers 5, 6, 7, 8, and 9, is positioned downstream of *Nfix* promoter 2 (Supplementary Figure S1D). Analysis of the chromatin state of *Nfix* enhancers revealed that enhancers located between *Nfix* promoters 1 and 2 became accessible from the P5 stage, in both myonuclei and SCs. Notably, these enhancers exhibited higher accessibility in quiescent SCs than in activated SCs, mirroring the chromatin accessibility profile observed for *Nfix* promoter 2 (Figure 2G,H). On the contrary, enhancers located downstream of the *Nfix* promoter 2 became accessible during the embryo-fetal switch in myonuclei and were also more accessible in activated SCs than quiescent ones, similar to *Nfix* promoter 1’s chromatin state (Figure 2G,H). Furthermore, enhancer–promoter contact prediction (ENC + EPD Enhc-Gene Track Encode3, UCSC) indicated that all *Nfix* intragenic enhancers interact with a specific region located between *Nfix* promoters 1 and 2 (Supplementary Figure S1D), suggesting that enhancer chromatin state may contribute to chromatin remodeling at the *Nfix* locus and potentially regulate Nfix expression during muscle development and regeneration. To determine whether the promoter-specific chromatin accessibility observed in mice is conserved in humans, we analyzed available human ATAC-seq datasets from quiescent SCs (GSE165414) and adult muscles (Gastrocnemius and Psoas, GSE184462 and GSM5214923, respectively) (Supplementary Figure S2). Consistent with our murine data, *Nfix* promoter 2 displayed higher chromatin accessibility in human SCs, whereas promoter 1 remained largely inaccessible. Conversely, adult muscles exhibited increased chromatin accessibility at promoter 1, suggesting a switch in promoter usage during myogenic differentiation (Supplementary Figure S2), and indicating that the promoter-specific chromatin accessibility profile observed in mice is conserved in human skeletal muscle.

Collectively, these findings identify *Nfix* promoter 1 as the “committed” promoter and *Nfix* promoter 2 as the “stem” promoter and highlight the potential role for intragenic enhancers in regulating chromatin architecture at the *Nfix* locus.

### 2.2. Dynamic Regulation of Nfix Expression During Skeletal Muscle Development and Regeneration

**Figure 3:**
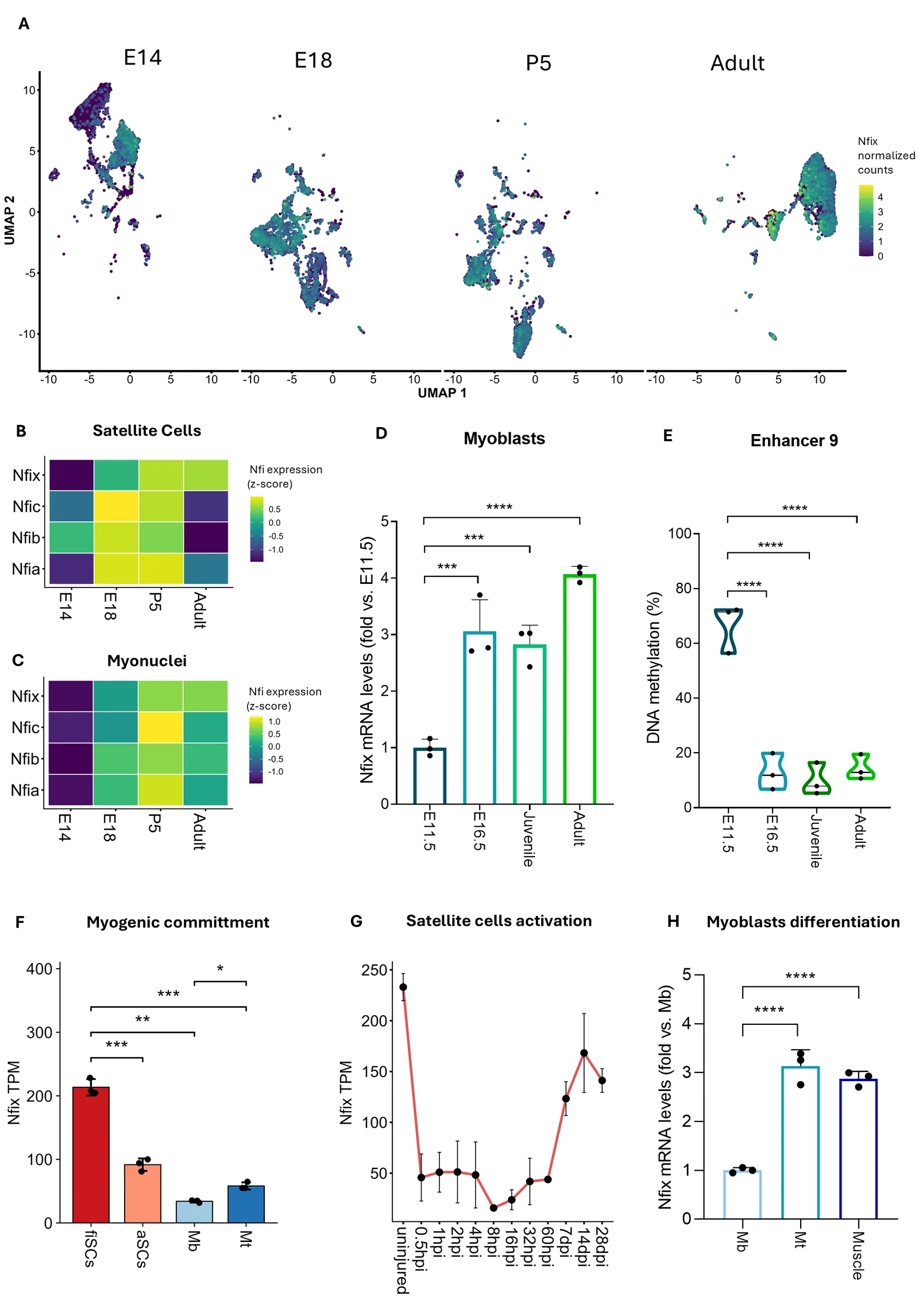
*Nfix* gene expression depends on developmental stages and cell type in skeletal muscle development and regeneration. (**A**) *Nfix* gene expression across skeletal muscle development represented through UMAP graphs (E14: embryonal; E18: fetal; P5: juvenile; Adult: adult). Expression data showed as Seurat-normalized counts. (**B**) *Nfi* gene expression during satellite cell and (**C**) myonuclei development. Data showed as row scaled expression (z-score). (**D**) Quantitative qRT-PCR analysis of *Nfix* expression in embryonal, fetal, juvenile, and adult myoblasts. Expression levels were normalized to Ribosomal Protein Subunit 29 (Rps29) mRNA levels and presented as mean ± SD. (**E**) Percentage of enhancer 9 DNA methylation according to MSRE-qPCR assay during skeletal muscle development. Values were normalized to non-digested samples. (**F**) Nfix TPM (Transcripts Per Million) expression during myogenic commitment and (**G**) satellite cell activation. (**H**) Quantification through qRT-PCR of *Nfix* gene expression during myoblast differentiation (Mbs: myoblasts; Mts: myotubes; Muscle: tibialis anterior). Expression levels were normalized to Ribosomal Protein Subunit 29 (Rps29) mRNA levels and presented as mean ± SD; one-way ANOVA: *: *p* < 0.05; **: *p* < 0.01; ***: *p* < 0.001; ****: *p* < 0.0001, *n* = 3.

Distinct chromatin accessibility profiles in *Nfix* promoters 1 and 2 in both SCs and myonuclei during myogenesis prompted us to decipher which *Nfix* promoter drives *Nfix* gene expression during skeletal muscle development and SC activation upon injury (Figure 1). Analysis of published snRNA-Seq (GSE211543) in embryonal (E14), fetal (E18), juvenile (P5), and adult (Adult) muscles revealed different *Nfi* family member expression based on cell type and developmental stage (Supplementary Figure S3A). Among the NFIs, we found that Nfix is the only NFI member whose expression increases during muscle development (Supplementary Figure S3B). Indeed, Nfix expression increases along with the development of adipocytes, mesenchymal cells, myonuclei, SCs, and tenocytes (Figure 3A and Figure S3C). Focusing on myogenic cell types (SCs and myonuclei), both E14 myonuclei and SCs do not express detectable levels of Nfi family members, while E18 myonuclei and SCs start to activate the expression *of Nfi* genes. Among the NFI members, Nfix progressively increases its expression in both P5 and adult SCs and myonuclei (Figure 3A–C and Figure S3D).

To validate these findings, we isolated embryonal (E11.5), fetal (E16.5), juvenile (2 weeks of age), and adult (12 weeks of age) myoblasts from murine muscles, and we assessed the Nfix mRNA levels. As expected, embryonal myoblasts expressed low levels of Nfix mRNA, while fetal, juvenile, and adult myoblasts exhibited significantly higher levels of the transcript (Figure 3D). We next asked whether Nfix expression is similarly regulated during adult muscle regeneration by analyzing Nfix mRNA levels from published bulk RNA-Seq data (GSE268313) from freshly isolated SCs (fiSCs), activated SCs (aSCs), myoblasts (Mbs), and myotubes (Mts). FiSCs express significantly higher levels of *Nfix*, compared to aSCs, Mbs, and Mts (Figure 3F). Notably, the *Nfix* gene expression progressively decreases in Mbs, whereas it is upregulated during skeletal muscle differentiation (from Mbs to Mts) (Figure 3F). Focusing on SC activation, we analyzed Nfix mRNA levels from published bulk RNA-Seq (GSE189073) on Pax7^GFP+^ cells isolated at different time points after murine muscle injury (uninjured, 0.5 hpi, 1 hpi, 2 hpi, 4 hpi, 8 hpi, 16 hpi, 32 hpi, 60 hpi, 7 dpi, 14 dpi, and 28 dpi). Assessment of mRNA levels revealed that the *Nfix* gene expression decreased starting from 0.5 hpi to 60 hpi. On the other hand, the *Nfix* gene expression was higher in uninjured SCs, and it was rescued at 7 dpi, 14 dpi, and 28 dpi (Figure 3G). Finally, we validated the increase in Nfix expression during myoblast differentiation. To this end, we assessed Nfix expression in SC-derived myoblasts, differentiated myotubes in culture, and in the *tibialis anterior* muscle. Our results showed that Nfix is upregulated during the transition from proliferating myoblasts (Mbs) to differentiated myotubes (Mts), and that its expression is maintained in *tibialis anterior* muscle (Figure 3H). Therefore, these findings suggest that Nfix acts as a marker of stemness in SCs and of proper myogenic differentiation in skeletal muscle.

To investigate the mechanisms underlying Nfix regulation during muscle development, we examined DNA methylation of *Nfix* enhancer 9, which has already been demonstrated to be differentially methylated in skeletal muscles [22]. To do that, we performed MSRE-qPCR of *Nfix* enhancer 9, and we observed a negative correlation between Nfix expression and DNA methylation levels at this regulatory element during muscle development, implying that DNA methylation might control the Nfix expression across muscle development by modulating enhancer 9 activity (Figure 3E).

To further characterize the epigenetic regulation of the *Nfix* locus, we analyzed DNA methylation profiles using whole-genome bisulfite sequencing (WGBS) data across skeletal muscle development (Embryonal hindlimb, [23]), adult muscle (*gastrocnemius*, [24]), and isolated adult SCs [25] (Supplementary Figure S4). This analysis revealed a highly dynamic, tissue-specific change in both key enhancers and alternative promoters. Among the enhancers, enhancer 3 (positioned between promoters 1 and 2) undergoes demethylation in both the *gastrocnemius* and SCs, suggesting its transition to an active or permissive chromatin state in adult tissues. Conversely, enhancer 9 undergoes a marked demethylation from the Embryonal hindlimb to the *gastrocnemius* (in line with the progressive decrease observed during development via MSRE-qPCR) but retains high methylation levels in SCs (Supplementary Figure S4A,B).

Strikingly, this enhancer dynamic is mirrored by a mutually exclusive epigenetic switch between alternative promoters 1 and 2 (Supplementary Figure S4A,C). *Nfix* promoter 1 is highly methylated in the embryo and in SCs, but completely loses methylation in the *gastrocnemius*, pointing to a strict epigenetic repression in stem cells. Conversely, *Nfix* promoter 2 exhibits low methylation levels in SCs but becomes highly methylated in the Gastrocnemius tissue (Supplementary Figure S4A,C). This distinct methylation pattern strongly suggests stage-specific promoter usage, where *Nfix* promoter 2 appears epigenetically poised to drive expression in adult stem cells, whereas *Nfix* promoter 1 is potentially reserved for adult muscles.

Overall, these findings suggest that a dynamic change in DNA methylation at regulatory enhancers and alternative promoters contributes to the stage-specific regulation of Nfix expression during skeletal muscle development and regeneration.

### 2.3. Dynamic Nfix Promoter Usage Shapes Isoform Expression During Skeletal Muscle Development and Differentiation

**Figure 4:**
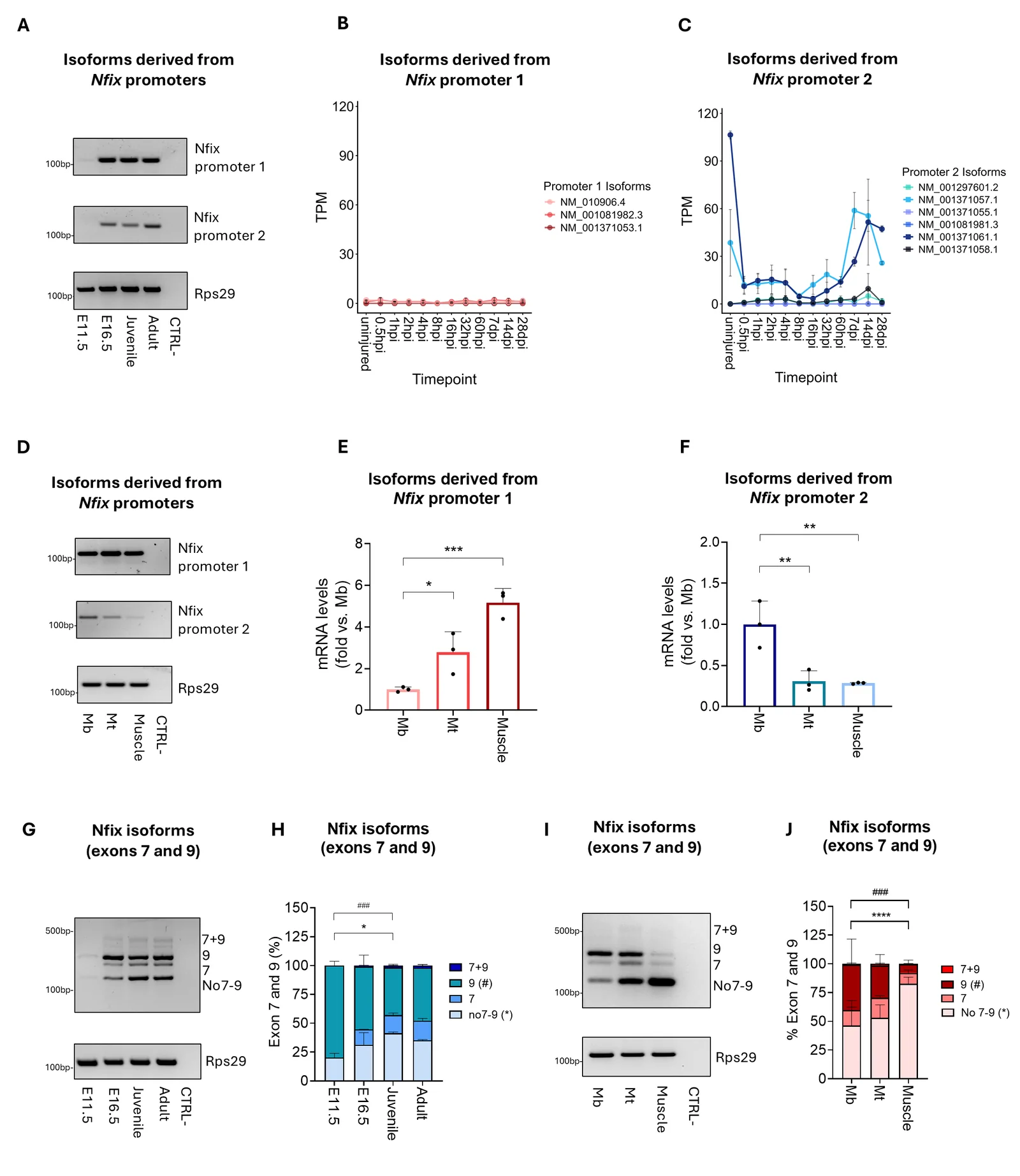
Nfix isoforms derived from *Nfix* promoters 1 and 2 affect the *Nfix* gene expression in skeletal muscle. (**A**) Agarose gel of PCR products of isoforms transcribed from *Nfix* promoters 1 and 2 during muscle development (E11.5: embryonal; E16.5: fetal). (**B**,**C**) Level of transcripts derived from *Nfix* promoters 1 (**B**) and 2 (**C**) from GSE211543 dataset and expressed in TPM. (**D**) Agarose gel of PCR products derived from isoforms transcribed from *Nfix* promoters 1 and 2 during muscle differentiation (Mbs: myoblasts; Mts: myotubes; Muscle: *tibialis anterior*). (**E**) Quantification through qRT-PCR of isoforms derived from promoter 1 and (**F**) promoter 2 during muscle differentiation. Expression levels were normalized to Ribosomal Protein Subunit 29 (Rps29) mRNA levels. (**G**) Agarose gels depicting PCR products of Nfix isoforms related to presence and absence of exons 7 and 9 during muscle development, and (**H**) muscle differentiation. (**I**) Percentage quantification of exon 7 and 9 presence during muscle development and (**J**) differentiation. (Data are presented as mean ± SD; one-way ANOVA: *: *p* < 0.05; **: *p* < 0.01; ***: *p* < 0.001; ****: *p* < 0.0001, #: *p* < 0.05; ###: *p* < 0.001, *n* = 3).

In light of the dynamic regulation of Nfix expression during myogenesis and muscle regeneration, we investigated the activity of *Nfix* promoters and their corresponding transcripts in skeletal muscle (Figure 1). At first, we analyzed the expression of the Nfix isoforms during skeletal muscle development and myoblast differentiation. As expected, *Nfix* promoters 1 and 2 were the most transcriptionally active, as indicated by the presence of isoforms originating from both promoters. In contrast, isoforms derived from *Nfix* promoters 3, 4, and 5 were barely detectable in skeletal muscle (Figure 4A,B and Figure S5A,B). During muscle development, embryonal myoblasts did not express isoforms derived from either *Nfix* promoter 1 or promoter 2, whereas fetal, juvenile, and adult myoblasts expressed isoforms derived from both promoters (Figure 4A). In adulthood, *Nfix* promoter 2 became transcriptionally active upon SC activation, particularly giving rise to two quiescent-relevant Nfix isoforms (NM_001371057.1 and NM_001371061.1) that mirrored the Nfix expression patterns observed during SC activation (GSE211543) (Figure 4B,C).

Further analysis of Nfix transcript expression during the muscle differentiation process (myoblasts, differentiated myotubes, *tibialis anterior* muscle) indicates that *Nfix* promoters 1 and 2 play opposite roles. Specifically, the expression of isoforms derived from *Nfix* promoter 2 decreased during myoblast differentiation, while isoforms originating from *Nfix* promoter 1 gradually increased, suggesting that *Nfix* promoters are differentially regulated and contribute distinctively to muscle differentiation (Figure 4D–F).

To get more insights into Nfix transcript diversity in skeletal muscles, we examined the inclusion of alternatively spliced exons 7 and 9, which encode part of the C-terminal domain of the NFIX protein. Our analysis revealed that in skeletal muscle, there is a different distribution of exon 7 and exon 9 usage within Nfix transcripts. Indeed, isoforms lacking both exons, as well as those containing either exon 7 or exon 9 alone, were differentially expressed across muscle development and differentiation, whereas isoforms containing both exons were not detected in skeletal muscle. Interestingly, during muscle development and myogenic differentiation, isoforms lacking both exons 7 and 9 progressively increased, whereas isoforms containing only exon 9 decreased (Figure 4G–J). Overall, these findings reveal a complex pattern of Nfix isoform expression in skeletal muscle, characterized by the presence of transcripts lacking exons 7 and 9, as well as transcripts containing either exon 7 or exon 9.

### 2.4. Distinct Nfix Isoforms Differentially Regulate Myoblast Proliferation and Fusion

Given the dynamic expression of Nfix isoforms during muscle development and differentiation, we investigated the role of exons 7 and 9 in regulating myogenic behavior (Figure 1).

To this end, we expressed Nfix isoforms either lacking or containing exons 7 and/or 9 in *Nfix*^−/−^ juvenile SC-derived myoblasts. After validating the proper expression of Nfix isoforms in each cell line (Supplementary Figure S6A,B), we examined myoblasts’ proliferative abilities and myogenic properties.

**Figure 5:**
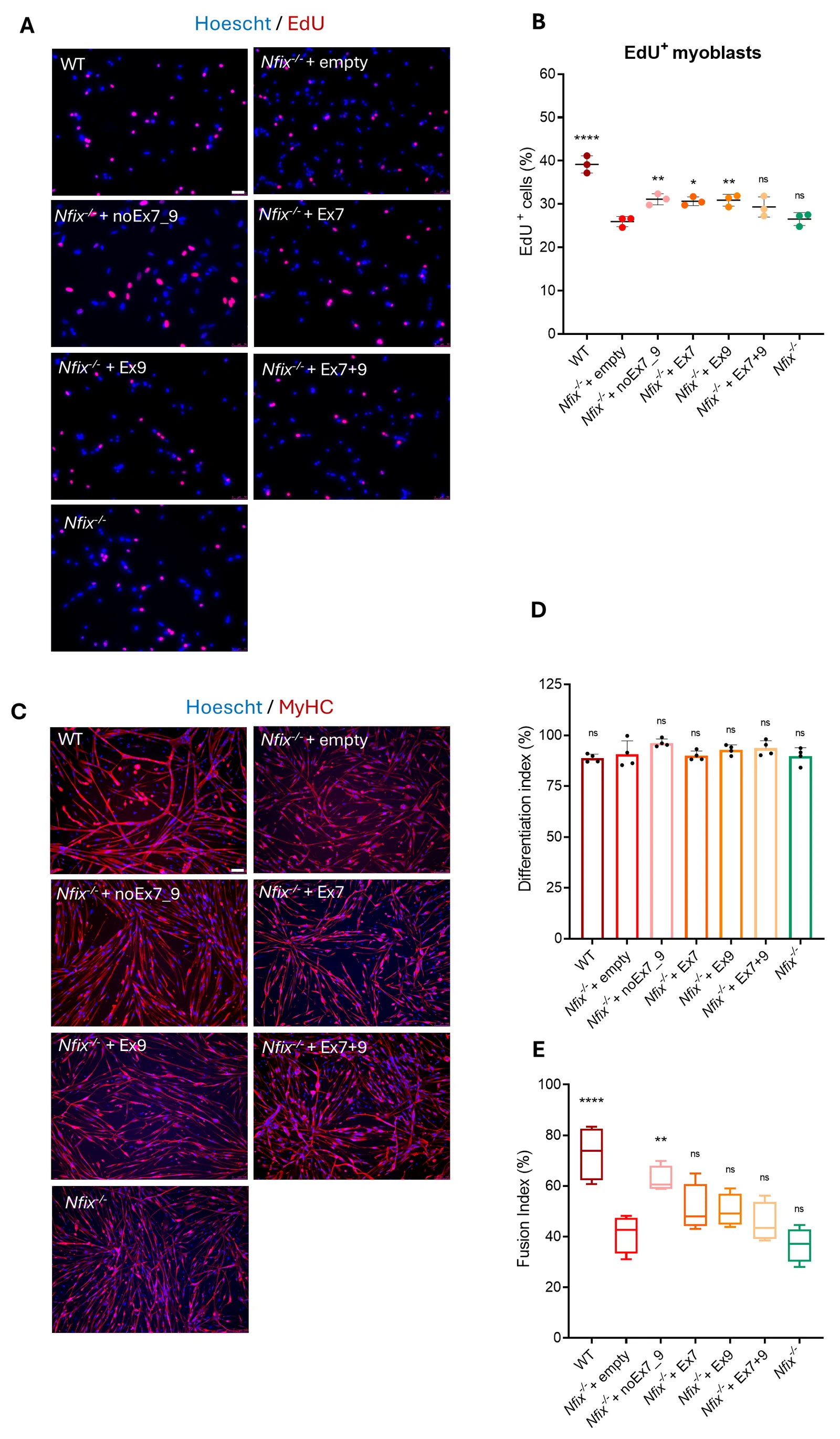
Nfix isoforms have specific activity in skeletal muscle. (**A**) EdU staining for *Nfix*^−/−^, *Nfix*^−/−^ + isoforms, and WT myoblasts (scale bar = 50 µm). (**B**) Quantification of EdU+ *Nfix*^−/−^, *Nfix*^−/−^ + isoforms, and WT myoblasts. (**C**) MyHC staining for *Nfix*^−/−^, *Nfix*^−/−^ + isoforms, and WT myotubes (scale bar = 100 µm). (**D**) Assessment of differentiation and (**E**) fusion indexes in *Nfix*^−/−^, *Nfix*^−/−^ + isoforms, and WT myotubes. Differentiation index was counted as MyHC+ nuclei divided by the total number of nuclei, while fusion index was calculated as the ratio of the number of nuclei contained in myotubes with three or more nuclei to the total number of nuclei. Data are presented as mean ± SD; one-way ANOVA: *: *p* < 0.05, **: *p* < 0.01; ****: *p* < 0.0001, *n* = 3 for EdU; *n* = 4 for MyHC (empty: empty plasmid; noEx7_9: lacking both exons 7 and 9; Ex7: carrying only exon 7; Ex9: carrying only exon 9; Ex7 + 9: carrying both exons 7 and 9), ns, not significant.

To assess the impact of Nfix isoforms containing different combinations of exons 7 and 9 on myoblast proliferation, we performed an EdU incorporation assay in the different cell lines. *Nfix*^−/−^ myoblasts displayed reduced proliferation compared to WT cells. Re-expression of most Nfix isoforms, particularly those lacking both exons 7 and 9 or containing either exon 7 or exon 9 alone, significantly increased myoblast proliferation. In contrast, isoforms containing both exons 7 and 9 did not affect myoblasts’ proliferative rates (Figure 5B).

To assess the contribution of Nfix isoforms to myogenic differentiation, myoblasts expressing the different exon 7/9 combinations were induced to differentiate and analyzed by MyHC staining. Thus, we quantified differentiation and fusion indexes, observing that *Nfix*^−/−^ myotubes showed reduced fusion ability compared to WT ones (Supplementary Figure S6D). Interestingly, myotubes expressing isoforms lacking both exons 7 and 9 rescued myotube fusion properties, supporting the relevance of these isoforms for skeletal muscle differentiation (Figure 5C,D and Figure S6C).

Overall, these findings indicate that distinct Nfix isoforms differentially regulate myogenic function: isoforms lacking both exons 7 and 9 or containing either exon alone promote myoblast proliferation, while isoforms lacking both exons 7 and 9 additionally enhance myogenic fusion.

### 2.5. Distinct Transcription Factor Networks Regulate Nfix Promoters During Skeletal Myogenesis

Nfix expression has been shown to exert detrimental effects in Muscular Dystrophies (MDs) [11,13], suggesting that its negative modulation could represent a promising therapeutic strategy. In addition, our findings indicate that *Nfix* promoter activity and isoform expression are dynamically regulated across distinct myogenic states. Thus, the identification of pathways controlling Nfix expression in different myogenic contexts may lay the groundwork for the development of Nfix-based therapeutic approaches.

**Figure 6:**
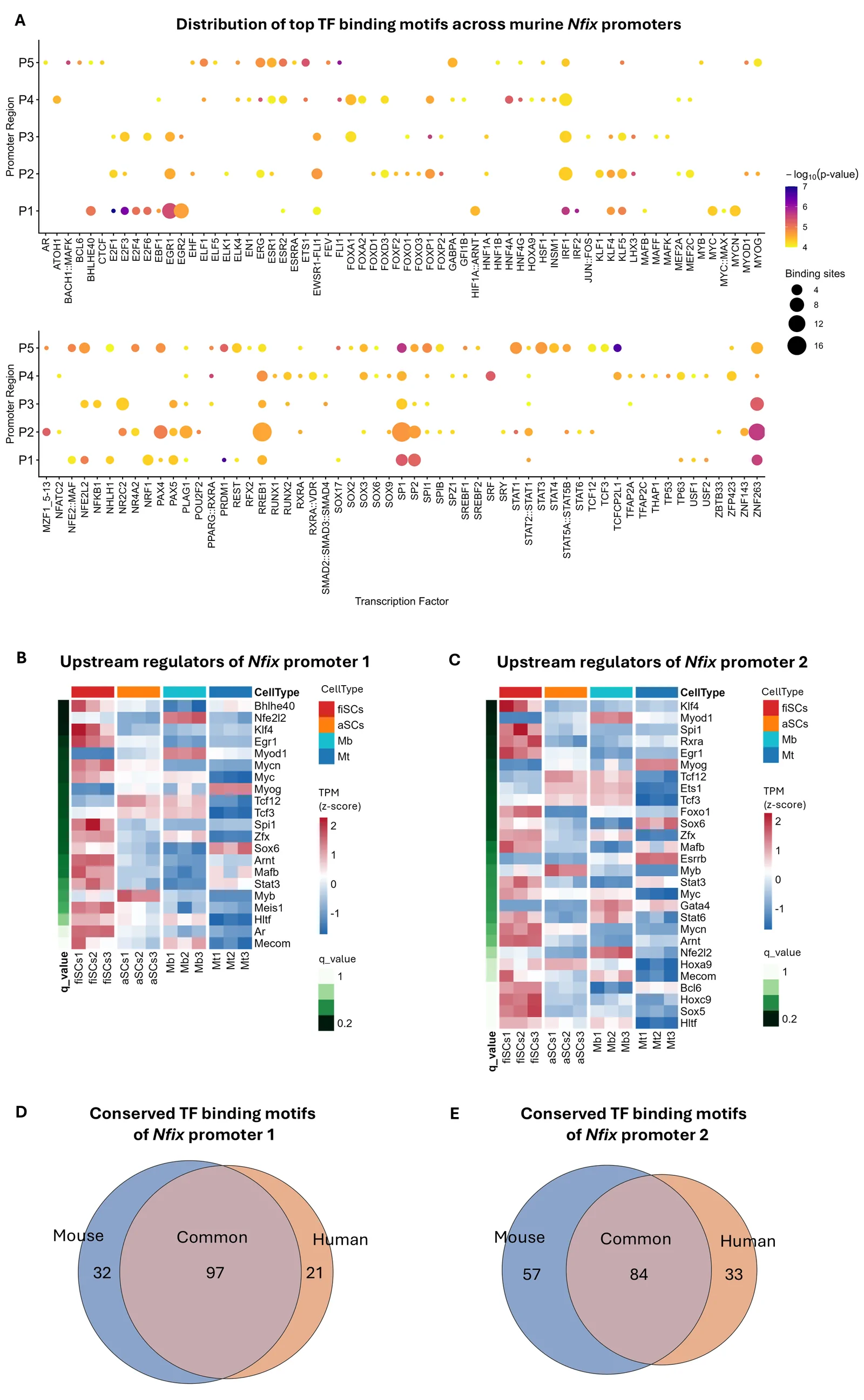
Upstream transcription factors binding *Nfix* promoters in murine and human contexts. (**A**) Distribution of top (*p* < 0.0001) TF-binding motifs across murine *Nfix* promoters (P1: promoter 1; P2: promoter 2; P3: promoter 3; P4: promoter 4; P5: promoter 5). (**B**) TPM of upstream regulators of *Nfix* promoter 1. (**C**) Same as (**B**) for *Nfix* promoter 2 in skeletal muscle during SC activation (fiSCs-aSCs) and myoblast differentiation (Mbs-Mts). (**D**) Percentage of TF motif conservation between murine and human *Nfix* promoter 1. (**E**) Same as (**D**) for *Nfix* promoter 2.

Therefore, we aimed to understand molecular pathways affecting *Nfix* promoter usage across various myogenic conditions (Figure 1). To do that, we investigated the upstream transcriptional networks that may regulate Nfix expression in skeletal muscle, analyzing *Nfix* promoter sequences with FIMO (MEME Suite [26]) and the JASPAR TF motif database. TF-binding motifs in the different *Nfix* promoters were identified in murine promoter sequences (Figure 6A and Figure S7A,B). FIMO and JASPAR analyses identified the TF-binding motifs of FoxD1, Irf2, Srf, and Stat6, which were specifically present in the distinct *Nfix* promoters, whereas TFs such as Irf1, Rreb1, and Plag1 were commonly detected in all the *Nfix* promoters (Figure 6A and Figure S7A,B, Supplementary Table S3). Gene ontology analysis of transcription factors associated with the different *Nfix* promoters showed distinct functional enrichments, supporting the existence of promoter-specific regulatory programs controlling Nfix expression. In particular, TFs predicted to bind promoters 1, 2, and 3 were enriched for pathways associated with organ morphogenesis, skeletal muscle development and differentiation, and miRNA-mediated regulatory processes, implying their involvement in distinct cellular and developmental contexts (Supplementary Figure S8).

To identify relevant transcriptional regulators of *Nfix* promoters during SC activation (fiSCs-aSCs) and muscle differentiation (Mbs-Mts), we integrated genes whose expressions were highly correlated with Nfix (GSE268313), with transcription factor binding predictions obtained from FIMO analyses. *Nfix* promoter 1 was associated with a more restricted set of putative transcription factors than promoter 2. TFs including MYCN, EGR1, KLF44, MAFB, MYOG, MYOD1, and SOX6 were detected in both promoters (Figure 6B,C). In contrast, BHLHE40, MEIS1, and MYB were selectively associated with promoter 1, whereas SPI1, ETS1, FOXO1, HOXC9, HOXA9, GATA44, and SOX5 were specific for promoter 2 (Figure 6B,C). Notably, ChIP-seq datasets confirmed the binding of MYOD1 and MYOG to both promoters, consistent with their predicted shared regulatory role, whereas BHLHE40 and MEIS1 binding was validated only at *Nfix* promoter 1, supporting their promoter-specific regulatory activity (Supplementary Figure S9) [27,28,29]. Collectively, these findings indicate that distinct yet partially overlapping transcription factor networks regulate *Nfix* promoters during skeletal muscle stem cell activation and differentiation.

Finally, given the detrimental role of Nfix in MDs, we aimed to identify regulatory pathways controlling the *Nfix* gene expression that are conserved between murine and human contexts and that might be relevant for Nfix-based therapeutic approaches in MD patients. Therefore, we investigated the conservation of murine *Nfix* promoters within the human genome. Three murine *Nfix* promoters were conserved in humans and displayed substantial preservation of TF-binding profiles (Nfix promoter 1: 64.7%, promoter 2: 48.3%, and promoter 3: 52.9%) (Figure 6D,E and Figure S7C). These findings suggest that the regulatory architecture controlling Nfix expression is conserved in humans, supporting the potential involvement of promoter-specific Nfix regulation in human muscle pathophysiology. In conclusion, we identified distinct promoter-specific transcriptional networks regulating Nfix expression during skeletal muscle stem cell activation and differentiation.

### 2.6. NFIX Orchestrates Transcriptional Programs Controlling Skeletal Muscle Structure and Metabolism

Our findings suggest that distinct signaling pathways may differentially regulate *Nfix* promoter activity, providing a potential framework for the development of NFIX-targeted therapeutic approaches. However, the molecular programs governed by NFIX have not yet been fully characterized. To define the molecular pathways controlled by NFIX, we performed RNA-seq in primary WT and *Nfix*^−/−^ myotubes (Figure 1).

**Figure 7:**
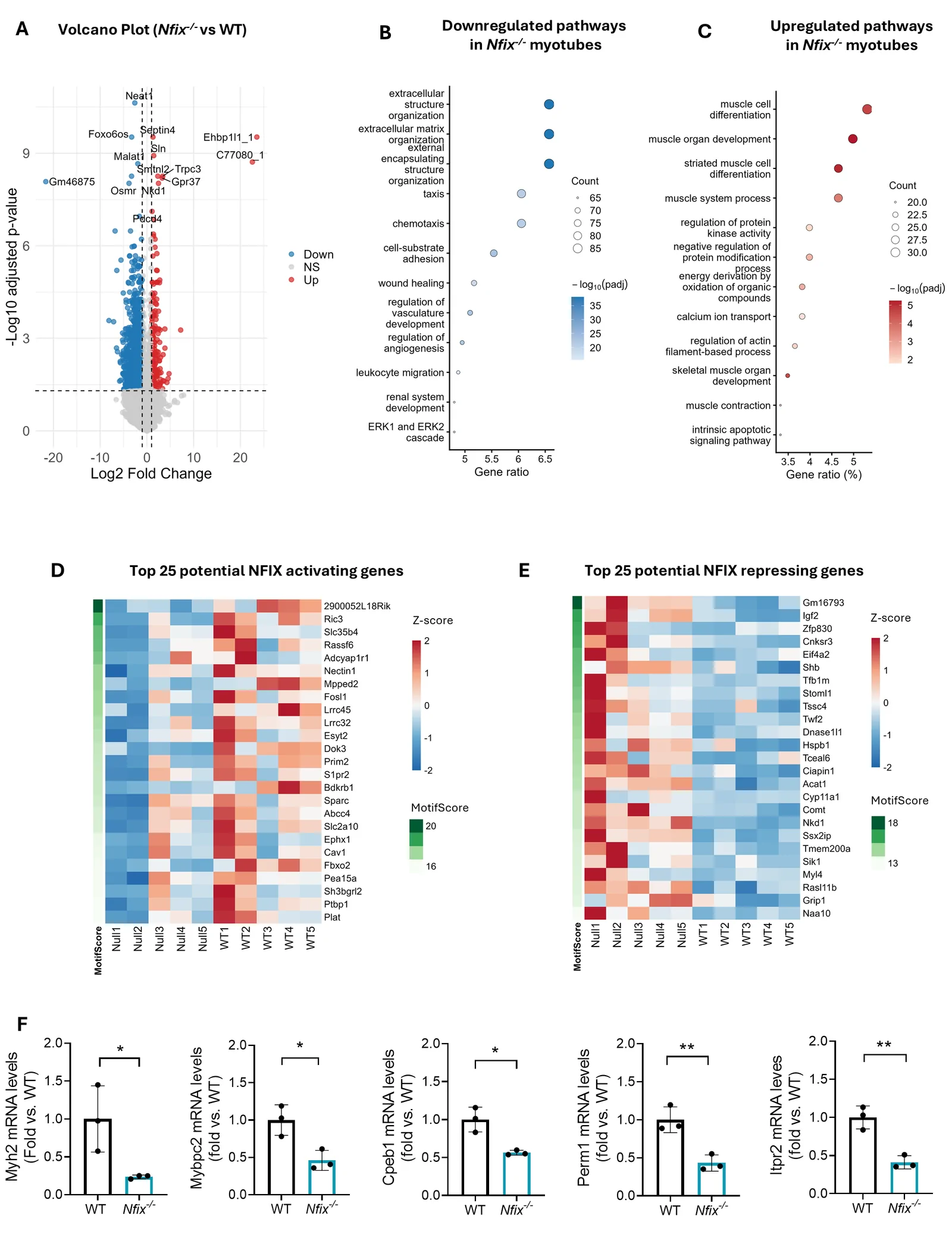
Potential Nfix target genes and their pathways. (**A**) Volcano plot derived from differentially expressed gene analysis between *Nfix*^−/−^ and WT myotubes (blue: downregulated genes, red: upregulated genes). (**B**) Gene ontology enrichment computed with enrichGO of downregulated and (**C**) upregulated genes. Terms are ranked according to the gene count ratio. (**D**) Heatmap showing variance-stabilizing transformed (VST) counts of potential NFIX activating genes. (**E**) Same as (**D**) for repressing genes. Genes are listed based on motif score. (**F**) mRNA levels in WT and *Nfix*^−/−^ myotubes of Myh2, Mybpc2, Cpeb1, Perm1, and Itpr2. Expression levels were normalized to Ribosomal Protein Subunit 29 (Rps29) mRNA levels. (data are presented as mean ± SD; *t*-test: *: *p* < 0.05; **: *p* < 0.01; *n* = 3).

Differential gene expression analysis revealed 2255 misregulated genes (padj < 0.05): 678 genes were upregulated in *Nfix*^−/−^ myotubes, whereas 1577 genes were downregulated (Figure 7A and Figure S10, Supplementary Table S1). To characterize the biological processes affected by NFIX loss, we performed a gene ontology enrichment analysis on the complete set of differentially expressed genes using the clusterProfiler and enrichGO (4.20.0) packages. Among the pathways significantly downregulated in *Nfix*^−/−^ myotubes, extracellular matrix organization, chemotaxis, and angiogenesis-related processes emerged, highlighting a central role for Nfix in coordinating the interaction between differentiating myofibers and their surrounding microenvironment [30]. Conversely, pathways upregulated in *Nfix*^−/−^ myotubes further emphasized the broad regulatory function of NFIX in skeletal muscle biology. The absence of NFIX was associated with altered expression in genes involved in muscle cell differentiation and muscle organ development, together with pathways linked to oxidative metabolism, calcium ion transport, and actin filament organization (Figure 7B,C).

Since NFIX loss altered the expression of genes associated with these processes, we next sought to identify potential direct NFIX target genes. To identify potential direct NFIX target genes among the differentially expressed genes, we searched for the NFIX binding motif within the promoters (−1000/+100 bp from the TSS) of differentially expressed genes. Among the 2255 differentially expressed genes, 666 contained at least one high-confidence NFIX binding site within their promoter, suggesting that they may represent direct NFIX target genes. Interestingly, 26% of potential NFIX target genes (179/666) were upregulated in *Nfix*^−/−^ myotubes, whereas 74% (487/666) of them were found to be downregulated (Figure 7D,E, Supplementary Table S2). Finally, based on previous studies implicating NFIX in skeletal muscle structural and metabolic programs [2,13], we assessed the expression of selected NFIX target genes in WT and *Nfix*^−/−^ myotubes. We therefore focused on NFIX target genes involved in muscle structure and metabolism, including *Myh2*, *Mybpc2*, *Cpeb1*, *Perm1*, and *Itpr2*. The expression of these genes was significantly reduced in *Nfix*^−/−^ myotubes (Figure 7F), further supporting the role of NFIX in the transcriptional regulation of skeletal muscle structural and metabolic genes [2,13]. Overall, these findings proved that NFIX is a key regulator of skeletal muscle metabolism and structural organization during myogenic differentiation.

## 3. Discussion

NFIX is recognized as an important regulator of skeletal muscle homeostasis, playing essential roles during fetal myogenesis and adult muscle regeneration [2,3]. In addition, modulation of Nfix expression has been associated with beneficial effects in Muscular Dystrophies [13]. However, the molecular mechanisms governing Nfix transcriptional regulation, promoter usage, and isoform expression remain poorly understood. This lack of fundamental knowledge hampers a comprehensive understanding of how NFIX activity is regulated in physiological and pathological conditions, thereby limiting the development of effective therapeutic strategies for NFIX-related disorders. Therefore, defining the transcriptional landscape governing Nfix expression is essential to elucidate the molecular mechanisms underlying its regulation. To address this gap, we investigated the transcriptional and epigenetic mechanisms regulating Nfix expression in skeletal muscle.

### 3.1. Nfix Promoter Switch Governs Stem-to-Committed Myogenic States

**Figure 8:**
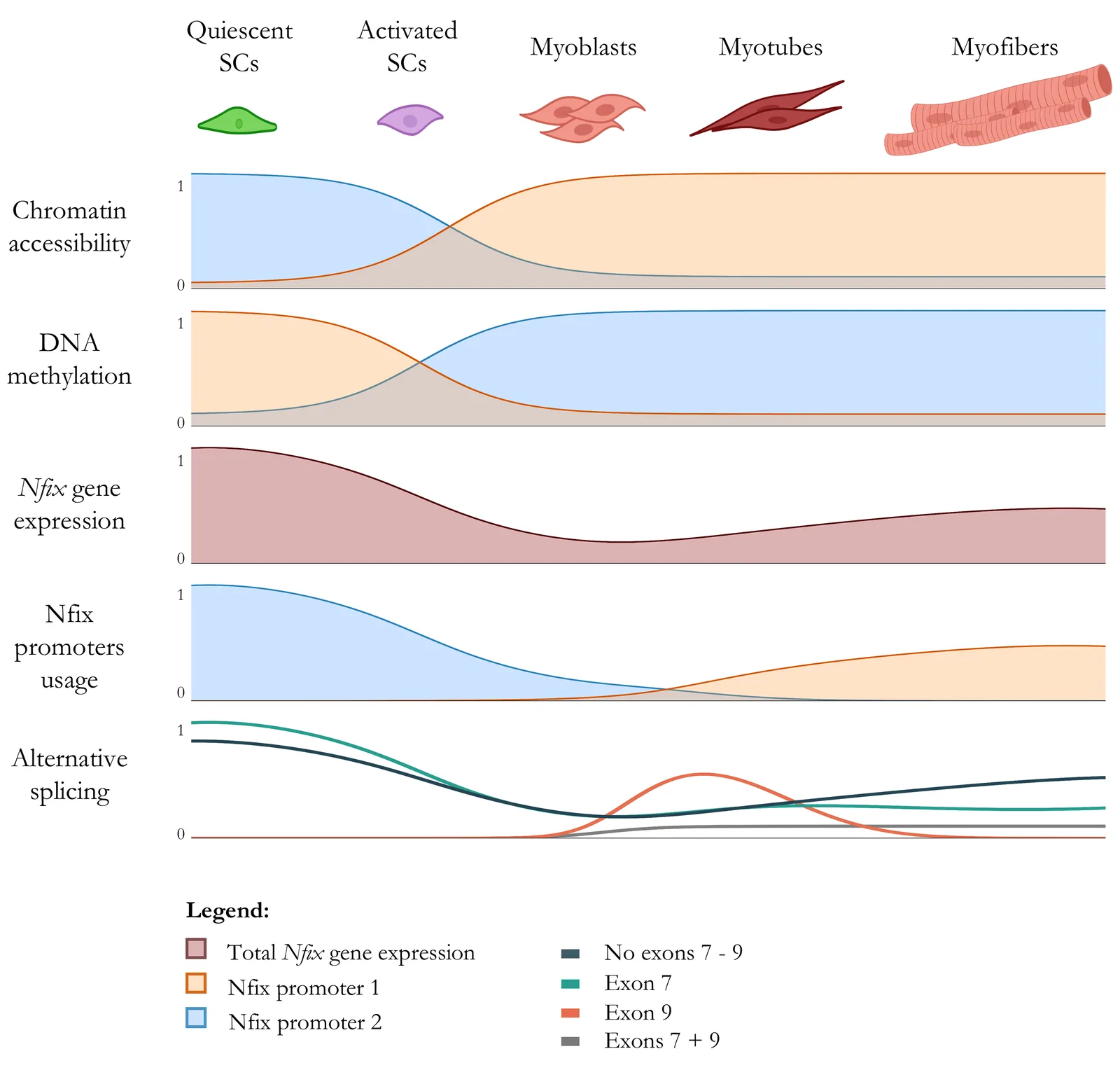
Model depicting the regulatory mechanisms governing Nfix expression during skeletal muscle differentiation. This model summarizes the major findings of the present study, highlighting the multilayered regulation of Nfix during skeletal muscle differentiation. It illustrates the coordinated interplay between chromatin accessibility, DNA methylation, promoter-specific transcript isoform expression (promoter usage), and alternative splicing that shapes the dynamic regulation of Nfix from quiescent satellite cells to mature myofibers. Values are shown on a relative scale, where 0 represents the minimum level and 1 the maximum level.

We observed that the *Nfix* gene is differentially regulated during skeletal muscle development as well as during adult muscle regeneration through *Nfix* promoter 1 and 2 accessibility, revealing two distinct types of *Nfix* promoters (Figure 8). *Nfix* promoter 2 functions as a “stem” *Nfix* promoter in quiescent SCs, whereas *Nfix* promoter 1 acts as a “committed” *Nfix* promoter during myoblast commitment. These findings suggest that Nfix expression is sustained by promoter-specific regulatory programs that are selectively engaged according to SC state. These data were supported by the specific presence of TF-binding motifs in promoter 1 (BHLHE40, MEIS1, and MYB) or promoter 2 (SPI1, ETS1, FOXO1, HOXC9, HOXA9, GATA4, and SOX5), indicating that different pathways might modulate Nfix expression. Interestingly, during muscle development, both *Nfix* promoters become accessible from the fetal phase during myonuclei development, implying that chromatin remodeling occurs in the transition between primary and secondary myogenesis.

### 3.2. Dynamic DNA Methylation Drives an Epigenetic Switch at Nfix Promoters and Enhancers

To decipher mechanisms affecting the Nfix chromatin change and thus the *Nfix* gene expression activation from the embryonal to fetal phase, we focused on DNA methylation because the *Nfix* gene has already been shown to be differentially methylated during development and in relation to different cell types [22,31,32].

Whole-genome bisulfite sequencing (WGBS) revealed that the progressive demethylation of enhancer 9 during skeletal muscle development is accompanied by a reciprocal methylation switch at the two alternative *Nfix* promoters. During the transition from embryonic tissue (Embryonic hindlimb) to adult muscle (gastrocnemius), *Nfix* promoter 1 becomes progressively demethylated, whereas *Nfix* promoter 2 acquires DNA methylation. Together with our transcriptomic analysis showing a progressive increase in Nfix-promoter-1-derived isoforms during myogenic differentiation, these findings support a model in which *Nfix* promoter 1 becomes the predominant driver of Nfix expression in differentiated skeletal muscle.

In contrast, adult SCs displayed the opposite methylation profile, with *Nfix* promoter 2 remaining largely unmethylated while *Nfix* promoter 1 retained high methylation levels. Consistent with the enrichment of *Nfix*-promoter-2-derived isoforms in quiescent SCs, these data suggest that *Nfix* promoter 2 represents the preferential promoter sustaining Nfix expression in the adult stem cell compartment. Notably, enhancer 9 remained highly methylated in SCs, whereas enhancer 3 was demethylated, raising the possibility that these enhancers cooperate with the alternative promoters to fine-tune Nfix expression in a cell-type-specific manner.

Together, these findings support a model in which DNA methylation orchestrates stage- and cell-type-specific Nfix expression by regulating alternative promoter usage, with *Nfix* promoter 1 preferentially driving transcription in differentiated myofibers and *Nfix* promoter 2 sustaining Nfix expression in adult SCs.

This model is consistent with previous studies demonstrating that DNA methylation is a central mechanism regulating Nfix expression not only in skeletal muscle but also across multiple tissues [19,31,32,,33]. Moreover, our findings suggest that, in addition to the established role of JUNB [9], epigenetic regulation through enhancer remodeling and alternative promoter usage represents an additional layer of control governing Nfix expression during the transition from primary to secondary myogenesis (Figure 8).

### 3.3. Alternative Splicing of Exons 7 and 9 Regulates Myoblast Proliferation and Fusion

In addition, our study revealed that Nfix expression is strictly linked to the abundance of exons 7 and 9 in Nfix transcripts in skeletal muscle (Figure 8). Exons 7 and 9, which belong to the C-terminal transactivation and/or repression domain of the NFIX protein, appear to exert specific regulatory functions. We discovered that Nfix isoforms lacking both exons 7 and 9 can rescue the myotube fusion gap observed in the *Nfix*^−/−^ myotubes. Interestingly, this effect was not observed with the other exon combinations, implying that the NFIX protein lacking exons 7 and 9 might interact with specific cofactors able to promote pro-differentiation programs. Notably, the abundance of isoforms lacking both exons 7 and 9 increases during muscle development and regeneration, supporting a role for these isoforms in myogenic commitment.

### 3.4. Distinct Transcription Factors Dictate Promoter-Specific Nfix Regulation

Since NFIX has a detrimental role in Muscular Dystrophies, we sought to identify transcription factors and pathways controlling its expression, with the aim of supporting the development of NFIX-targeted therapeutic approaches. Our work highlighted possible upstream regulators of the *Nfix* gene. The shared presence of TF-binding motifs for key myogenic regulators, including MYOD1, MYOG, SOX6, and MYCN, in both promoters suggests the existence of a core transcriptional program driving Nfix expression during muscle stem cell activation and differentiation. At the same time, the selective enrichment of distinct TFs on promoters 1 and 2 points to specialized regulatory modules that may fine-tune Nfix expression according to specific developmental stages or cellular contexts. These findings provide a first framework of promoter-specific transcriptional regulators potentially controlling Nfix expression in distinct myogenic contexts.

### 3.5. NFIX Coordinates Myotube Structure, Calcium Dynamics, and Metabolism

Finally, to define the molecular pathways controlled by NFIX we performed RNA-seq on WT and *Nfix*^−/−^ myotubes. Transcriptomic analysis revealed that NFIX target genes are involved in extracellular matrix organization, angiogenesis-related processes, muscle organ development and differentiation, mitochondrial activity, calcium ion transport, and sarcomere organization. These findings suggest that NFIX actively promotes extracellular matrix remodeling and pro-angiogenic signaling, two key processes required for proper muscle maturation and tissue organization [30]. Furthermore, our results position NFIX as a central transcriptional regulator coordinating both structural and metabolic programs required for myotube maturation and functional muscle development. These data were supported by downregulated expression of *Myh2* and *Mybpc2* genes in *Nfix*^−/−^ myotubes, supporting a role for NFIX in the regulation of genes structurally involved in the generation of fast myofibers. These outcomes are consistent with previous studies showing that NFIX dictates fiber-type switching during muscle development [34], and that its silencing promotes a more oxidative phenotype by converting fast-twitch glycolytic fibers into slow-twitch oxidative ones [13]. Additionally, Cpeb1 mRNA levels were reduced in *Nfix*^−/−^ myotubes. CPEB1 is an RNA-binding protein, and its binding to the Myod1 transcript is fundamental to promote SC activation and proliferation [35]. Thus, the decrease in *Cpeb1* expression due to the absence of NFIX might explain the delay of muscle regeneration observed in injured *Nfix*^−/−^ mice, highlighting NFIX’s relevance in the control of muscle regeneration timing. Furthermore, we demonstrated that NFIX also acts on calcium signaling. Indeed, Itpr2 (the calcium channel that transfers calcium from the endoplasmic reticulum to mitochondria) decreased in the absence of NFIX, supporting the fact that NFIX has a primary role in muscle metabolism and mitochondrial activity. These observations were also supported by reduced Perm1 mRNA levels in *Nfix*^−/−^ myotubes, which is a central factor for muscle metabolism [36].

## 4. Materials and Methods

### 4.1. Animal Work

All mice were kept under pathogen-free conditions with a 12 h/12 h light/dark cycle. All of the procedures on animals conformed to Italian law (D. Lgs n. 2014/26, as the implementation of 2010/63/UE) and were approved by the University of Milan Animal Welfare Body and by the Italian Ministry of Health. Female mice were mated with males (2:1) and examined every morning for copulatory plugs. The day on which a vaginal plug was seen was designated as gestation day 0.5 (E0.5). The following mouse lines were used: *Myf5*^GFP-P/+^ (CD1) [37] and *Nfix*^−/−^ (C57BL/6J-129S4) (obtained from Prof. Richard M. Gronostajski, University of Buffalo, NY, USA) [38].

### 4.2. Cell Isolation and Culture

*Myf5*^GFP-P/+^ embryonic muscles [37] were isolated at E11.5, and fetal muscles at E16.5. Muscles were mechanically and enzymatically digested for 30 min at 37 °C under agitation with 1.5 mg/mL Dispase (Gibco, Thermo Fisher Scientific, Waltham, MA, USA, 17105041), 0.15 mg/mL collagenase (Sigma-Aldrich, St. Louis, MO, USA, C9263), and 0.1 mg/mL DNase I (Roche, Basel, Switzerland, 11284932001), as previously described [39]. The dissociated cells were filtered and collected in Dulbecco’s modified Eagle’s medium (DMEM) with high glucose (EuroClone, Milan, Italy, ECB7501L), 20% fetal bovine serum (Thermo Fisher Scientific, Waltham, MA, USA, A5256701), 2 mM EDTA, and 20 mM HEPES. The *Myf5*^GFP-P/+^ myoblasts were sorted (BD Biosciences, FACSAria, Franklin Lakes, NJ, USA) and cultured in high-glucose DMEM, 20% horse serum (Gibco, Thermo Fisher Scientific, Waltham, MA, USA, 16050122), 2 mM L-glutamine (EuroClone, Milan, Italy, ECB3000D), 100 IU/mL penicillin, and 100 mg/mL streptomycin (EuroClone, Milan, Italy, ECB3001D), in collagen-coated plates.

Juvenile and adult SC-derived myoblasts were isolated from 14-day-old and 12-week-old WT and *Nfix*^−/−^ mice. All hindlimb muscles were collected from each mouse and placed into DMEM medium with 1% penicillin/streptomycin. Muscles were mechanically and enzymatically digested for 20 min at 37 °C under strong agitation with 1.5 mg/mL Dispase, 0.15 mg/mL collagenase, and 0.1 mg/mL DNase I in PBS 1× (EuroClone ECB4004L). After every digestion cycle, dissociated cells were collected in DF10 medium (DMEM, 10% FBS, 1% P/S, 1% L-Glutamine EuroClone ECB30000D) at 4 °C to inactivate the enzymes. Once all the digestion cycles had finished and all cells had been gathered in the same tubes, they were centrifuged at 300 *g* for 5 min at 4 °C. The pellet was resuspended in DF10 medium and filtered using the 70 μm nylon filter and then the 40 μm nylon filter. Then, the cell pellet was resuspended in MACS buffer (phosphate-buffered saline, pH 7.2, 0.5% bovine serum albumin, 2 mM EDTA) and a Satellite Cell Isolation Kit (Miltenyi Biotec, Bergisch Gladbach, Germany; 130-104-268) according to manufacturer’s instructions. The mixture was incubated at 4 °C for 15 min and then magnetically separated following the Miltenyi protocol. The flow-through, which contains the myogenic cells, was collected, centrifuged at 300 *g* for 5 min, and resuspended in proliferation medium for satellite cells (DMEM, 20% FBS, 10% Horse Serum, 1% P/S, 1% L-Glutamine, 0.1% Gentamycin (Sigma-Aldrich, St. Louis, MO, USA, G1397), 2.5 ng/mL β-FGF (PeproTech, Cranbury, NJ, USA, 100-18B), in gelatin-coated plates.

For cell transfection, *Nfix*^−/−^ myoblasts were transfected with the Lipofectamine 3000 transfection reagent (Invitrogen, Thermo Fisher Scientific, Waltham, MA, USA, L3000015). At 16 h post-transfection, the media was replaced, and at 24 h post-transfection, Puromycin Dyhidrochloride (1 µg/mL; Invitrogen, Thermo Fisher Scientific, Waltham, MA, USA, A1113803) was added to the media until the negative control completely died. Cells were then maintained in Puromycin Dyhidrochloride selection (0.5 µg/mL) for further experiments.

To perform in vitro differentiation of myoblasts into myotubes, cells were plated at 80% of confluence, and the day after, the media were replaced with differentiation medium and myotubes were analyzed at 3 days of differentiation (DMEM, 2% HS, 1% P/S, 1% L-Glutamine).

### 4.3. Plasmids

The plasmid combinations containing different combinations of exons 7 and 9 were as follows: pBiLenti-GFP (empty plasmid), pBiLenti-NFIX1HA-GFP (exon 7), pBiLenti-NFIX2HA-GFP (no exons 7 and 9), pBiLenti-NFIX6HA-GFP (exons 7 + 9), pBiLenti-NFIX8HA-GFP (exon 9). Plasmids were purchased from Genewiz. Then, for plasmid amplification and purification, each plasmid was transformed into the DH5α *E. coli* strain and purified through NucleoBond Xtra Maxi EF protocol (MACHEREY-NAGEL GmbH & Co., KG, Düren, Germany, 740424.50), according to manufacturer instructions.

### 4.4. Single-Nucleus RNA and ATAC Sequencing Analysis

Single-nucleus RNA expression and chromatin accessibility data generated using the 10X Genomics Chromium Single Cell Multiome ATAC + Gene Expression platform were obtained from the Gene Expression Omnibus (GEO) database as processed barcodes, features, and count matrices (GSE211542). Data were analyzed in R using the Seurat and Signac packages (version 5.3.1 and 1.16.0, respectively), enabling joint analysis of gene expression and chromatin accessibility measured in the same nucleus. Multimodal integration was performed using the weighted nearest neighbor (WNN) framework, with PCA applied to the RNA assay and latent semantic indexing (LSI) to the ATAC assay, followed by UMAP visualization and clustering. Cell identities were assigned based on canonical marker gene expression as previously reported [40]. Gene expression and chromatin accessibility at the *Nfix* locus were visualized using feature plots, violin plots, and aggregated accessibility profiles across developmental stages. Markers used to label cell populations in snRNA-Seq and chromatin coordinates used for snATAC-seq are listed in Supplementary File S1.

### 4.5. DNA Methylation Analysis by MSRE-qPCR and Whole-Genome Bisulfite Sequencing (WGBS)

Embryonal, fetal, juvenile, and adult genomic DNA was extracted from the cell pellet, incubating it overnight with Lysis buffer (1%SDS, 0.01 M Tris pH = 8, 1 mM EDTA pH = 8, 0.1 M NaCl in sterile water) and Proteinase K (1 mg/mL; Cell Signaling Technology, Danvers, MA, USA, #10012)) at 55 °C. The following day, the isopropanol–ethanol protocol was used to obtain genomic DNA, which was resuspended in sterile water. Then, genomic DNA was digested with 10 U of methylation-sensitive restriction enzyme SmaI (Promega Corporation, Madison, WI, USA, R6121) at 25 °C for 2 h. Thereafter, digested and non-digested (negative control) genomic DNA was used to perform qRT-PCR as described in paragraph 4.9. A genomic region in the *Nfix* gene without SmaI restriction enzyme digestion sites was used as a positive control of the assay. Whole-genome bisulfite sequencing (WGBS) methylation-rate BigWig files (mm10) corresponding to the Embryonic hindlimb (GSE158647), the adult *gastrocnemius* (GSE210391), and adult satellite cells (GSE171604) were retrieved from ChIP-Atlas. DNA methylation profiles were visualized in the UCSC Genome Browser. Mean DNA methylation levels were calculated as the average methylation percentage of all CpG sites within the genomic regions of interest (*Nfix* enhancers and promoters) and used for comparative analyses.

### 4.6. ATAC-Seq Data Processing

ATAC-seq BigWig signal tracks from the dataset GSE189044 were analyzed across a time course following injury (uninjured; 0.5–60 h post-injury; 7–28 days post-injury). Analyses were performed in R using the packages GenomicRanges (1.58.0), rtracklayer (1.66.0), pheatmap (1.0.13), and ggplot2 (4.0.3). Genomic regions of interest within the *Nfix* locus (mm10 reference genome) are listed in Supplementary File S1, including nine putative enhancers and five promoter regions on chromosome 8. ATAC-seq signal was extracted from BigWig files using rtracklayer, restricting the import to regions of interest. For each region and time point, the signal was quantified as the mean of overlapping scores, generating a region-by-time matrix. Signal values were log-transformed using log_2_(1 + x).

Human ATAC-seq BigWig signal tracks from publicly available datasets were analyzed to evaluate the conservation of NFIX promoter accessibility patterns in human skeletal muscle. ATAC-seq datasets from human satellite cells and differentiated skeletal muscle tissues were visualized at the NFIX locus using the hg38 reference genome. Signal tracks were inspected together with MACS2-called accessible regions to assess chromatin accessibility at NFIX promoter 1 and promoter 2. ATAC-seq signal intensity was evaluated across the NFIX promoter regions to compare accessibility profiles between satellite cells and differentiated muscle tissues.

### 4.7. RNA-Seq Processing and Expression Analysis

Generation of RNA-seq data between *Nfix*^−/−^ and WT myotubes was performed as described: 2-week-old *Nfix*^−/−^ and WT SC-derived myoblasts were extracted as described in Section 4.2 and maintained in cell culture. Then, 10^5^ myoblasts were plated in each well of a 12-well multiwell, and the day after, the media were replaced, adding 2%HS medium. After 3 days of differentiation, myotubes were harvested, and RNA was extracted according to the TRIzol Reagent protocol (Invitrogen, Life Technologies Corporation, Carlsbad, CA, USA; 15596026, as described in Section 4.13).

RNA sequencing was performed by Novogene GmbH (Martinsried, Munich, Germany). Paired-end RNA-seq reads were aligned against the mouse reference genome (mm10) with RSEM using STAR as the mapping tool. Raw FASTQ files were processed with *rsem-calculate-expression* with *gzip-compressed input files* enabled. Gene-level expected counts and TPM generated by RSEM were imported into R for downstream analyses. Lowly expressed genes were filtered out by retaining only genes with counts > 10 total across all samples. Differential expression analysis was performed using the DESeq2 package (1.46.0). Batch effects related to sequencing day were included in the DESeq2 design formula and corrected for visualization using limma (3.62.2). Variance-stabilizing transformation (VST) was applied for PCA and visualization analyses. Differentially expressed genes were identified using adjusted *p* < 0.05 (Benjamini–Hochberg correction). Functional enrichment analyses for Gene Ontology Biological Processes (GO-BP) were performed using the clusterProfiler (4.14.0) and org.Mm.eg.db packages (3.20.0). Results were visualized using ggplot2 (4.0.3) and enrichplot (1.26.6).

Already published RNA-seq datasets (GSE189074 and GSE268313) were processed as follows: raw RNA-seq reads were downloaded as compressed FASTQ files from public repositories based on their SRA accession numbers. Transcript quantification was performed with RSEM using the STAR aligner in single- or paired-end mode against a pre-indexed mouse mm10 RefSeq transcriptome reference. Gene- and transcript-level expression values were obtained as TPM or counts, and used for downstream analyses. Expression matrices were analyzed in R using the packages ggplot2 (4.0.3), dplyr (1.2.0), and pheatmap (1.0.13).

### 4.8. Detection of Potential NFIX Target Genes

Potential NFIX target genes were identified by integrating differential expression analysis with transcription factor motif scanning. Differentially expressed genes (adjusted *p* < 0.05) between *Nfix*^−/−^ and WT myotubes were converted from gene symbols to Entrez IDs using the org.Mm.eg.db annotation database. Transcription start site (TSS) coordinates were obtained from the TxDb.Mmusculus.UCSC.mm10.knownGene annotation package (3.10.0), and promoters were defined as the genomic regions spanning from −1000 bp upstream to +100 bp downstream of the TSS. Promoter sequences were extracted from the mouse reference genome (mm10) using the BSgenome.Mmusculus.UCSC.mm10 (1.4.0) package. NFIX position weight matrices (PWMs) were retrieved from the JASPAR2020 database using TFBSTools (1.44.0). Motif scanning was performed with the matchMotifs function from the motifmatchr (1.28.0) package using a significance threshold of *p* < 1 × 10^−4^. For each promoter region, the number of NFIX motif occurrences was calculated, and motif scores associated with each hit were extracted from the PWM match metadata. The maximum motif score detected within each promoter was retained as the representative NFIX binding score for that gene. Genes containing at least one NFIX motif site were classified as potential NFIX target genes. Motif occurrence counts and maximum motif scores were merged with DESeq2 (1.46.0) differential expression results and normalized expression values for downstream analyses and ranking of candidate NFIX-regulated genes.

### 4.9. Identification of Upstream Regulators of Nfix

To identify candidate transcriptional regulators of Nfix, transcription factor binding motifs were analyzed within the *Nfix* promoter regions. *Nfix* promoter sequences spanning −500 bp to +100 bp relative to the transcription start site were retrieved from mouse (mm10) and human (hg38) genomes. Motif scanning was performed using FIMO from the MEME Suite [26], with transcription factor binding profiles obtained from the JASPAR database. All predicted transcription factor binding motifs within the *Nfix* promoter were catalogued to generate an initial list of potential upstream regulators. To prioritize candidate regulators with potential functional relevance in muscle tissue, the list of transcription factors identified through motif scanning was integrated with Nfix-correlated genes for the Gene Expression Omnibus dataset GSE268313 (containing fiSC, aSC, Mb, and Mt samples). Nfix-correlated genes were obtained from the expression matrix of GSE268313 using TPM-normalized RNA-seq expression data across all samples. Lowly expressed genes were filtered by retaining genes with mean TPM ≥ 5, and expression values were log_2_-transformed before the analysis. Pearson correlation coefficients were calculated between Nfix expression and all the other genes, followed by Benjamini–Hochberg FDR correction for multiple testing. Genes with Nfix correlation indexes greater than 0.9 or less than −0.9 were considered as “highly-Nfix-correlated genes”. Transcription factors showing consistent expression patterns and significant positive or negative correlation with Nfix were considered candidate upstream regulators. The resulting set of candidate transcription factors was visualized and summarized using plots generated in R with the ggplot2 (4.0.3) and pheatmap (1.0.13) packages. TF conservation percentage was obtained by evaluating how many transcription factors were in common between human and murine conserved Nfix promoters.

To experimentally support the predicted transcription factor binding sites, candidate transcription factors (MYOD, MYOG, BHLHE40, and MEIS1) were further evaluated using publicly available ChIP-seq datasets retrieved from ChIP-Atlas. For selected transcription factors, both ChIP-seq signal tracks (BigWig files) and MACS2-called peak files were analyzed at the Nfix locus using the mm10 reference genome. Only high-confidence peaks generated by MACS2 (*q-value* < 0.00001) were considered for validation. ChIP-seq signal tracks and binding peaks were visualized together with *Nfix* promoter regions using the UCSC Genome Browser to determine whether candidate transcription factors showed enrichment at promoter 1 and/or promoter 2. This analysis was used to validate the promoter-specific binding profile of selected transcription factors identified through motif analysis and correlation-based prioritization.

### 4.10. Immunofluorescence of Cultured Cells

Cell cultures were fixed for 10 min at 4 °C with 4% paraformaldehyde in PBS, and were then permeabilized with 0.2% Triton X-100 (Sigma-Aldrich, St. Louis, MO, USA, T8787) and 1% bovine serum albumin (BSA; Sigma-Aldrich, St. Louis, MO, USA, A7906) in PBS for 30 min at room temperature. After permeabilization, the cells were treated with blocking solution (10% goat serum; Sigma-Aldrich, St. Louis, MO, USA, G9023) for 30 min at room temperature, and then incubated overnight at 4 °C with mouse anti-total MyHC [hybridoma MF20; 1:2; Developmental Studies Hybridoma Bank, University of Iowa, Iowa City, IA, USA (DSHB)] antibody. The day after, cells were washed twice with PBS, 1% BSA, and 0.2% Triton, and incubated for 45 min at room temperature with the secondary antibodies (1:250; Jackson ImmunoResearch, West Grove, PA, USA: goat anti-mouse 594, 92278) and Hoechst (1:500; Cell Signaling Technology, Danvers, MA, USA; 33342). Finally, the cells were washed twice with 0.2% Triton in PBS and mounted with Fluorescence Mounting Medium (Dako, Agilent Technologies, Santa Clara, CA, USA, S3023). Images were acquired with a fluorescence microscope (DMI6000B; Leica Microsystems GmbH, Wetzlar, Germany) equipped with a digital camera (DFC365FX; Leica Microsystems GmbH, Wetzlar, Germany), and channels were merged as necessary using ImageJ (1.54t). Cell counting, differentiation, and fusion index analysis were performed using ImageJ. The differentiation index was calculated as the number of MyHC+ nuclei divided by the total number of nuclei, whereas the fusion index was calculated as the ratio of the number of nuclei within myotubes containing three or more nuclei to the total number of nuclei.

### 4.11. EdU Staining of Cultured Cells

For EdU incorporation, 5 × 10^4^ myoblasts were plated in 35 mm Petri dishes. The day after, cells were exposed for 1 h to 10 μm EdU (Invitrogen, Thermo Fisher Scientific, Waltham, MA, USA; C10339). The cells were then fixed with 4% PFA for 15 min at RT, washed twice with 3% BSA in PBS, and permeabilized with 0.5% Triton X-100 (Sigma-Aldrich, St. Louis, MO, USA, T8787) in PBS for 20 min. Primary antibody (Invitrogen, Thermo Fisher Scientific, Waltham, MA, USA; C10339) was incubated for 30 min at room temperature. Cells were then washed again with 3% BSA in PBS and stained with Hoescht (Cell Signaling Technology, Danvers, MA, USA; 33342) for nuclei visualization detection. Images were acquired with a fluorescence microscope (DMI6000B; Leica Microsystems GmbH, Wetzlar, Germany) equipped with a digital camera (DMI6000B; Leica Microsystems GmbH, Wetzlar, Germany), and channels were merged as necessary using ImageJ. Cell counting was performed using ImageJ.

### 4.12. Protein Extraction and SDS-PAGE Western Blotting

Protein extracts were obtained from cultured myoblasts lysed using RIPA buffer [10 mM Tris-HCl (pH 8.0), 1 mM EDTA, 1% Triton-X, 0.1% sodium deoxycholate, 0.1% sodium dodecylsulphate (SDS), and 150 mM NaCl in deionized water] for 30 min on ice. RIPA buffer was supplemented with protease and phosphatase inhibitors. After lysis, the samples were centrifuged at 10,000 rpm for 10 min at 4 °C, and the supernatants were collected for protein quantification (DC Protein Assay; Bio-Rad Laboratories, Hercules, CA, USA). For Western blotting, 50 μg of total protein extract was denatured with SDS Page Loading sample buffer (100 mM Tris pH 6.8, 4% SDS, 0.2% Bromophenol blue, 20% Glycerol, and 10 mM dithiothreitol) and heated at 95 °C for 5 min. Then, denatured protein samples were loaded on 10% SDS acrylamide gel to perform electrophoresis running and then blotted to a 0.45 μm nitrocellulose transfer membrane (Thermo Fisher Scientific, Waltham, MA, USA; 88018). Afterwards, the membrane was blocked in 5% Milk in TBST 1X (NaCl 150 mM, Tris-HCl 20 mM, Tween20 0.02%) for 1 h and then primary antibodies dissolved in 5% Milk TBST 1X or Signal Boost (Calbiochem, Merck KGaA, Darmstadt, Germany; 407207) were incubated O/N at 4 °C in agitation. We used the following primary antibodies and dilutions: rabbit anti-Nfix (1:2500, Geneka Biotechnology Inc., Montreal, QC, Canada; 16021118). The day after, blots were washed with TBST 1X and incubated with secondary antibodies (1:10,000, anti-mouse or anti-rabbit, IgG-HRP, Bio-Rad Laboratories, Hercules, CA, USA; 1706516, 1706515) at room temperature for 45 min in 5% Milk TBST 1X. Protein bands were revealed through ECL detection reagent (GeneSpin, PDS standard), and images were acquired using the ChemiDoc MP System (Bio-Rad Laboratories, Hercules, CA, USA).

### 4.13. RNA Extraction, RNA Retrotranscription, and qRT-PCR

Total RNA was isolated from muscle sections or myogenic cells through the TRIzol Reagent protocol (Invitrogen, 15596026). The obtained RNA pellet was allowed to air dry for about 10 min, avoiding its complete dryness and then resuspended in 10–15 μL of Nuclease-free water (Invitrogen, Thermo Fisher Scientific, Waltham, MA, USA; 4387936). RNA samples were quantified through NanoDrop Spectrophotometer (Implen GmbH, Munich, Germany) and stored at −80 °C or retrotranscribed. Total RNA was retrotranscribed to complementary DNA (cDNA) with iScript Reverse Transcription Supermix (Bio-Rad Laboratories, Hercules, CA, USA; 1708840) following the manufacturer’s protocol. The cDNA obtained was diluted in RNase-free water and stored at −20 °C or used for qRT-PCR. The real-time PCR was performed using SYBR Green Supermix (Bio-Rad Laboratories, Hercules, CA, USA; 1725274). Relative mRNA expression levels were normalized to the Ribosomal Protein Subunit 29 (Rps29) gene expression levels. PCR product from cDNA was resolved on 3.1% agarose gel.

### 4.14. Quantification and Statistical Analysis

All data are expressed as mean ± SD. Graphs were obtained using GraphPad Prism software (8.0.2) and analyzed with the normalization test and the appropriate statistical test, as indicated in figure legends, together with sample size. We used a two-tailed *t*-test or one-way ANOVA to compare two groups or more than two groups, respectively. *: *p* < 0.05, **: *p* < 0.01; ***: *p* < 0.001; ****: *p* < 0.0001, confidence interval 95%, alpha level 0.05.

## 5. Conclusions

Collectively, our findings highlight NFIX as a central regulator of skeletal muscle identity, integrating transcriptional, epigenetic, and post-transcriptional mechanisms to coordinate muscle development and regeneration. Specifically, state-specific promoter usage, DNA methylation, and alternative splicing finely tune Nfix expression (Figure 8), while distinct upstream networks and downstream targets dictate myofiber fate. The clinical relevance of these regulatory mechanisms (particularly at the promoter level) is supported by human genetic data; for instance, the SNP rs148229471 within the human NFIX core promoter is clinically classified as an “NFIX-related disorder” (ClinVar Accession ID: VCV000707679). Deciphering how this regulatory network becomes altered in disease may open new avenues for precision therapies targeting NFIX-related muscular disorders, including Muscular Dystrophies.

## Data Availability

The original data presented in this study are openly available in the GEO DataSets at https://www.ncbi.nlm.nih.gov/gds (accessed on 5 August 2026), GSE333416.

## Supplementary Materials

The following supporting information can be downloaded at: https://www.mdpi.com/article/10.3390/ijms27167367/s1.

## Abbreviations

The following abbreviations are used in this manuscript:

aSCs	Activated Satellite Cells
Dpi	Days Post Injury
Eno3	β-enolase
EPD	Eukaryotic Promoter Database
fiSCs	Freshly Isolated Satellite Cells
Hpi	Hours post injury
MCKs	Muscle Creatine Kinases
MRFs	Muscle Regulatory Factors
MSRE	Methylation Sensitive Restriction Enzyme
Mstn	Myostatin
Myf5	Myogenic Factor 5
Myf6	Myogenic Factor 6
MyHC	Myosin Heavy Chain
Myod1	Myogenic Determinant Factor 1
NFIA	Nuclear Factor I A
NFIB	Nuclear Factor I B
NFIC	Nuclear Factor I C
NFIX	Nuclear Factor I X
Pax7	Paired Homeobox Domain 7
Pax3	Paired Homeobox Domain 3
PKCθ	Protein Kinase C θ
qPCR	Quantitative Polymerase Chain Reaction
SCs	Satellite Cells
TPM	Transcripts Per Million
