# Supplementary material for: Dynamic NFIX Promoter Usage Shapes Stem and Committed Myogenic States
Source: Int J Mol Sci. 2026 Aug 18;27(16):7367. doi: 10.3390/ijms27167367 (PMC13513291; doi:10.3390/ijms27167367)
Supplement: Supplementary file 1 [file ijms-27-07367-s001.zip › Supplementary File S1. Information of the study.pdf]

Markers for each cell population of snRNA-Seq and snATAC-Seq along skeletal muscle development

Myonuclei = "Ttn","Trdn","Mylk4","Neb","Dmd"
Mesenchymal = "Tmeff2","Ebf2","Abca8a","Col3a1","Fap"
SCs = "Pax7","Pde1c","Map2","Fgf12","Hs6st2"
Myoblasts = "Myo16","Megf10","Tmem178b","Slc24a3","Dpp6"
Tenocytes = "Col11a1","Col1a1","Col1a2","Itgbl1","Mkx"
Endothelial = "Flt1","Ptprb","Prex2","Pecam1","Mecom"
Macrophages = "F13a1","Mctp1","Mrc1","Slc9a9","Rbpj"
Smooth muscle = "Myh11","Itga1","Cacnb2","Dgkb","Kcnab1"
Adipocytes = "Pparg","Cidec","Prkar2b","Pde3b","Acsl1"

Nfix promoters and specific isoforms in relationship with exons 7 and 9 combinations of the *Nfix* gene

Nfix promoter	Transcript name	Exons 7 - 9
Nfix promoter 1	NM_010906.4	Lacks both Exons 7 and 9
Nfix promoter 1	NM_001081982.3	Only exon 7
Nfix promoter 1	NM_001371053.1	Both exons 7 and 9
Nfix promoter 2	NM_001297601.2	Lacks both Exons 7 and 9
Nfix promoter 2	NM_001371058.1	Only exon 7
Nfix promoter 2	NM_001371061.1	Lacks both Exons 7 and 9
Nfix promoter 2	NM_001371055.1	Only Exon 9
Nfix promoter 2	NM_001371057.1	Only exon 7
Nfix promoter 2	NM_001081981.3	Both exons 7 and 9
Nfix promoter 3	NM_001371060.1	Only exon 7
Nfix promoter 3	NM_001371062.1	Only exon 7
Nfix promoter 4	NM_001371059.1	Only exon 7
Nfix promoter 4	NM_001371054.1	Both exons 7 and 9
Nfix promoter 4	NM_001371056.1	Only exon 7
Nfix promoter 4	NM_001371052.1	Both exons 7 and 9
Nfix promoter 5	NM_001371063.1	Only exon 7

Chromatin coordinates of Nfix promoters and enhancers (Grc38/mm10):

Promoter 1: chr8, 84799000 - 84801000
Promoter 2: chr8, 84773500 - 84774250
Promoter 2: chr8, 84779400 - 84780800
Promoter 4: chr8, 84772450 - 84773000
Promoter 5: chr8, 84775500 – 84776100

Enhancer 1: chr8, 84792001 - 84797400
Enhancer 2: chr8, 84787201 - 84791200
Enhancer 3: chr8, 84784901 - 84786900

Enhancer 4: chr8, 84777201 - 84783400
Enhancer 5: chr8, 84769101 - 84771748
Enhancer 6: chr8, 84760101 - 84762100
Enhancer 7: chr8, 84757201 - 84759400
Enhancer 8: chr8, 84748401 - 84753800
Enhancer 9: chr8, 84737401 - 84747600

Gene Expression Omnibus Datasets used in this study

Assay	GSE	DOI
snATAC-Seq, myogenesis	GSE211542 (GSE211545)	10.1038/s41467-023-40073-8
snRNA-Seq, myogenesis	GSE211543 (GSE211545)	10.1038/s41467-023-40073-8
ATAC-Seq, SC activation (time points)	GSE189044 (GSE189074)	10.1016/j.isci.2022.104954
RNA-Seq, SC activation (time points)	GSE189073 (GSE189074)	10.1016/j.isci.2022.104954
RNA-Seq, muscle regeneration	GSE268313	10.1002/jcsm.13682
MYOD binding at Nfix promoters	GSE36024 (SRX142536)	10.1038/nature13992
MYOG binding at Nfix promoters	GSE36024 (SRX142514)	10.1038/nature13992
BHLHE40 binding at Nfix promoter 1	GSE125729 (SRX5299300)	10.1038/s41590-019-0382-5
MEIS1 binding at Nfix promoter 1	GSE33067 (SRX101594)	10.1093/nar/gks595
ATAC-Seq, Human Satellite cells	GSE165414	10.1016/j.scr.2021.102496
ATAC-Seq, Human Gastrocnemius	GSE184462	10.1016/j.cell.2021.10.024
ATAC-Seq, Human Psoas	GSM5214923	10.1038/nature13992
DNA methylation, Embryonic Hindlimb	GSE158647 (GSM4804953)	10.1038/s41419-021-03817-2
DNA methylation, Gastrocnemius	GSE210391 (GSM6429601	10.1152/physiolgenomics.00129.2022
DNA methylation, Satellite cells	GSE171604 (GSM5229302)	10.1038/s41467-023-36265-x

Primers used in this study

Assay	Target	Sequence
MSRE-qPCR	Enhancer 9	FOR: GCACTCTTGGGGCCAGGTC<br>REV: GCAGGGGGTGATGGAAGAGAC
MSRE-qPCR	Negative Control	FOR: AGAGACCTCGTCACCATCCC<br>REV: GCTCTCCATAGGTGACGGC
qPCR	Nfix	FOR: CTGGCTTACTTTGTCCACACTC<br>REV: CCAGCTCTGTCACATTCCAGAC
qPCR	RPS29	FOR: AGTCACCCACGGAAGTTCG<br>REV: AGCATGATCGGTTCCACTTG
PCR; qPCR	Nfix promoter 1 isoforms	FOR: ATGTACTCCCCGTACTGCCT<br>REV: CTCGTCCTTTGACATCCGCT
PCR; qPCR	Nfix promoter 2 isoforms	FOR: TCAGAATCAGGCGTTGGCTT<br>REV: CTCGTCCTTTGACATCCGCT
PCR	Exons 7 - 9	FOR: AGCAGCCAGTCTAGCGGATG<br>REV: GATCCGATGCTGACAAACCG
qPCR	Myh2	FOR: GCGAATCGAGGCCCAGAATA<br>REV: TTCTTTCACGGTCAGGGTCG
qPCR	Mybpc2	FOR: CTGCTAGGGCCTGGTTAGAG<br>REV: CCTTTTTGGCCGCTGGTTTA

qPCR	Cpeb1	FOR: CAGCAGGACGGTGTTTGTTG<br>REV: TCACACGACCAGACCCAATG
qPCR	Itpr2	FOR: ACAGCAACGTTATCCAACTGC<br>REV: CAGCATTCACATTATCACCCTCG
qPCR	Perm1	FOR: TCTGTACCTCCAGGGGGAAG<br>REV: CGGGGTTAGGAAGGGTGAAC
